# Histone deacetylase inhibition enhances extracellular vesicles from muscle to promote osteogenesis via miR-873-3p

**DOI:** 10.1038/s41392-024-01976-0

**Published:** 2024-09-30

**Authors:** Ming Chen, Yi Li, Mingming Zhang, Siliang Ge, Taojin Feng, Ruijing Chen, Junmin Shen, Ran Li, Zhongqi Wang, Yong Xie, Duanyang Wang, Jiang Liu, Yuan Lin, Feifan Chang, Junyu Chen, Xinyu Sun, Dongliang Cheng, Xiang Huang, Fanfeng Wu, Qinxiang Zhang, Pingqiang Cai, Pengbin Yin, Licheng Zhang, Peifu Tang

**Affiliations:** 1 Senior Department of Orthopedics, The Fourth Medical Center of Chinese PLA General Hospital, Beijing, China; 2 National Clinical Research Center for Orthopedics, Sports Medicine & Rehabilitation, Beijing, China; 3 The Department of Orthopedic Surgery, Second Affiliated Hospital of Harbin Medical University, Harbin, China; 4 Jiangsu Key Laboratory of Molecular Medicine, Medical School, Nanjing University, Nanjing, China; 5 Innovative Centre for Flexible Devices (iFLEX), Max Planck-NTU Joint Lab for Artificial Senses, School of Materials Science and Engineering, Nanyang Technological University, Singapore, Singapore

**Keywords:** Bone remodelling, Trauma

## Abstract

Regular physical activity is widely recognized for reducing the risk of various disorders, with skeletal muscles playing a key role by releasing biomolecules that benefit multiple organs and tissues. However, many individuals, particularly the elderly and those with clinical conditions, are unable to engage in physical exercise, necessitating alternative strategies to stimulate muscle cells to secrete beneficial biomolecules. Histone acetylation and deacetylation significantly influence exercise-induced gene expression, suggesting that targeting histone deacetylases (HDACs) could mimic some exercise responses. In this study, we explored the effects of the HDAC inhibitor Trichostatin A (TSA) on human skeletal muscle myoblasts (HSMMs). Our findings showed that TSA-induced hyperacetylation enhanced myotube fusion and increased the secretion of extracellular vesicles (EVs) enriched with miR-873-3p. These TSA-EVs promoted osteogenic differentiation in human bone marrow mesenchymal stem cells (hBMSCs) by targeting H2 calponin (CNN2). In vivo, systemic administration of TSA-EVs to osteoporosis mice resulted in significant improvements in bone mass. Moreover, TSA-EVs mimicked the osteogenic benefits of exercise-induced EVs, suggesting that HDAC inhibition can replicate exercise-induced bone health benefits. These results demonstrate the potential of TSA-induced muscle-derived EVs as a therapeutic strategy to enhance bone formation and prevent osteoporosis, particularly for individuals unable to exercise. Given the FDA-approved status of various HDAC inhibitors, this approach holds significant promise for rapid clinical translation in osteoporosis treatment.

## Introduction

For centuries, it has been widely recognized that engaging in physical activity can significantly lower the risk of various disorders, ranging from metabolic diseases to cancer.^1–3^ The major player responsible for physical activity is the skeletal muscles, which release proteins, metabolites, and extracellular vesicles (EVs) that take part in tissue communication and have numerous beneficial health effects on different organs and tissues.^4^ Most of these molecules produced by skeletal muscles are reliant on muscle contraction during physical activity.^5,6^ Unfortunately, not all individuals are able to engage in physical exercise or muscle contraction training, particularly those who are elderly or suffer from clinical conditions.^7,8^ Therefore, it is essential to search for additional ways to stimulate the muscles to secrete more beneficial biomolecules, in addition to discovering new factors released by muscles and clarifying their functions.

Epigenetic modifications, particularly histone acetylation and deacetylation, play crucial roles in exercise-induced gene expression.^9,10^ Acetylation modifies the nucleosome structure, enhancing the accessibility of transcriptional regulatory proteins to chromatin templates, thereby promoting transcriptional activity.^11,12^ Interestingly, a single bout of exercise has been shown to trigger increases in histone acetylation in human skeletal muscle. In contrast, histone deacetylases (HDACs) function by removing acetyl groups from histones, resulting in chromatin condensation and subsequent repression of gene transcription. It’s intriguing to note that during exercise, class IIa HDACs are exported out of the nucleus, potentially influencing the nature and function of nano-sized EVs released during muscle contractions.^9,13,14^ As such, disrupting the class II HDAC corepressor complex, using genetic or pharmacological strategies, may provide an alternative pathway to simulate exercise-like transcriptional responses in skeletal muscle and consequent modifications in EV output. This suggests that targeting HDACs could provide a way to recapitulate some aspects of the exercise adaptive muscle response, potentially providing treatment options for those who are unable to engage in physical activity. Yet, a comprehensive understanding of the effects of HDAC inhibition on the muscle’s secretome, and its subsequent impact on the target organ, requires further research.

Histone deacetylase inhibitors (HDACi) comprise an expanding class of drugs capable of inhibiting the enzymatic activity of various HDACs. Emerging evidence suggests that these epigenetic agents are not only effective in treating malignancies, inflammatory conditions, and degenerative disorders, but also show promise in addressing skeletal muscle diseases.^15–17^ Administering different HDAC inhibitors, including trichostatin A (TSA), valproic acid, and sodium butyrate, which are broad-spectrum inhibitors of class I and II HDACs, to wild-type myoblasts enhances myoblast fusion efficiency and promotes myogenic differentiation.^18,19^ Among the three compounds, Minetti et al. reported that TSA was the most well-tolerated HDAC inhibitor for long-term treatment, with no significant side effects or signs of toxicity, such as weight loss, hyperexcitability, or sudden death. Conversely, an increased incidence of mortality was observed in valproic acid- and phenylbutyrate-treated mice.^20^ These results encouraged us to select TSA as one of the most potent and broad-spectrum histone deacetylase inhibitors for extensive analysis of skeletal muscle exercise and epigenetic activity. Indeed, HDACs inhibitors have a long history of use in psychiatry and neurology as mood stabilizers and anti-epileptics.^21,22^ Recently, a pan-HDACs inhibitor has been approved by U.S. Food and Drug Administration (FDA) for the treatment of cutaneous T cell lymphoma (CTCL).^23^ In this study, we investigated the impact of a selective HDAC inhibitor, Trichostatin A (TSA), on human skeletal muscle myoblasts (HSMMs). We harvested EVs from TSA-treated HSMMs, and then administered them to mice with estrogen deficiency-induced osteoporosis to ascertain whether HDAC inhibition could spur the release of beneficial EVs from muscle cells. Moreover, TSA-EVs mimicked the osteogenic benefits of exercise-induced EVs, suggesting that HDAC inhibition can replicate exercise-induced bone health benefits. Our findings revealed that HDAC inhibition stimulates the release of EVs laden with miR-873-3p from HSMMs, which subsequently confer osteoporosis protection in mice. When transferred to human bone marrow mesenchymal stem cells (hBMSCs), these EVs target H2 calponin (*CNN2*), thereby fostering the osteogenesis process. This study presents a novel approach to mimic the benefits of exercise, highlighting the potential role of muscle-derived EVs in disease treatment. Furthermore, considering the existing FDA approval for HDAC inhibitors, our findings could significantly expedite the clinical translation of this therapeutic strategy.

## Results

### The effects of HDAC inhibition on human skeletal muscle myoblast function and EV secretion

**Fig. 1:**
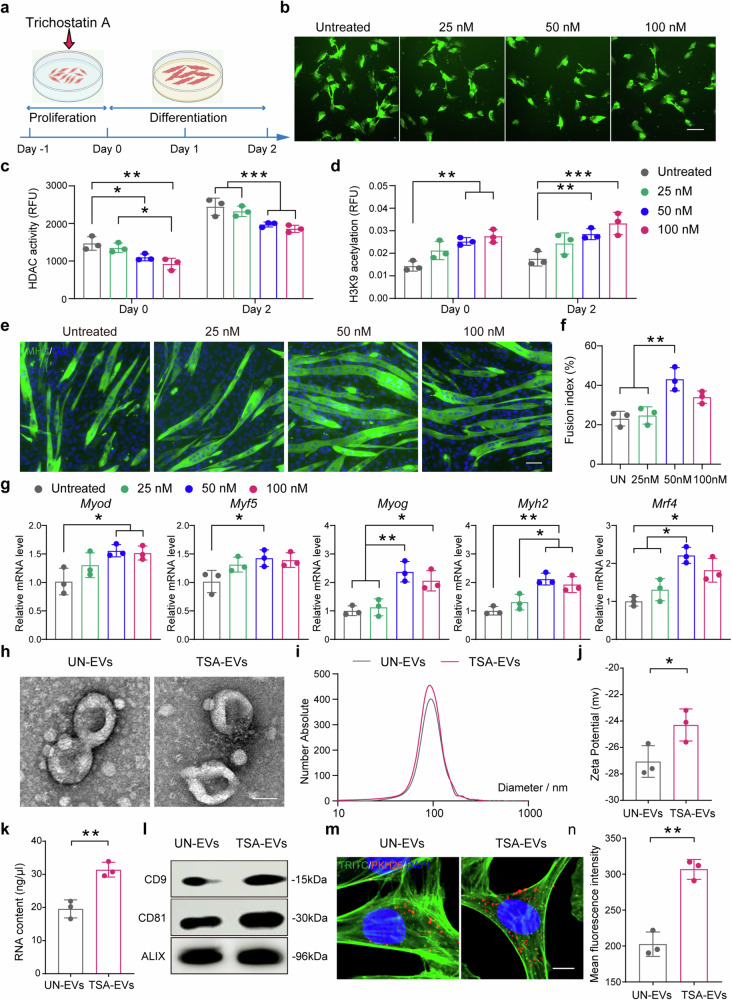
The effects of HDAC inhibition on human myoblasts functionality and EV secretion. **a** Graphical scheme of the experimental workflow. **b** Cell viability and morphology assessment by live/dead assay. Scale bar: 100 μm (*n* = 3 per group). **c**, **d** HDAC activity (**c**) and H3K9 histone acetylation (**d**) in a time-dose-dependent manner (*n* = 3 per group). **e**, **f** Myogenic differentiation of HSMMs measured by immunocytochemistry using an anti-MHC antibody (**e**) and the corresponding quantitative analysis (**f**). Scale bar: 50 μm (*n* = 3 per group). **g** Myogenic gene expression analysis in differentiating HSMMs using qPCR (*n* = 3 per group). qPCR results are presented as fold change relative to control. **h** TEM images of UN-EVs and TSA-EVs. Scale bar:50 nm. **i** Particle size distribution of isolated EV samples from NTA. **j** Zeta potential analysis. **k** EV RNA quantification. **l** Western blot analysis of EV markers. **m**, **n** Representative images (**m**) and quantifications (**n**) of the uptake of UN-EVs and TSA-EVs by hBMSCs. Scale bar: 10 μm (*n* = 3 per group). All data are presented as the mean ± SD. *p < 0.05; **p < 0.01; ***p < 0.001. Statistical significance was determined by one-way ANOVA test (**c**, **d**, **f**, **g**) and two-tailed Welch’s t test (**j**, **k**, **n**)

HDACs are key regulators of histone acetylation. HDAC inhibitors block the removal of acetyl groups from lysine residues on histone proteins, which leads to the relaxation of chromatin structure and activation of gene expression.^21,24,25^ To determine the effects of HDAC inhibition on human skeletal muscle myoblast (HSMM) epigenetic activity and biological function, HSMMs were exposed to TSA, an organic compound, in a dosage-dependent manner (Fig. 1a). Myoblast viability, cellular morphology, and metabolic activity were assessed (Fig. 1b). In assessing epigenetic function, TSA treatment led to a dose-dependent reduction in HDAC activity, with significant decreases observed at 50 nM and 100 nM compared to untreated cells (Fig. 1c). Moreover, TSA induced a dose-dependent increase in histone H3K9 acetylation, with 50 nM and 100 nM concentrations significantly enhancing acetylation levels relative to untreated cells (Fig. 1d). To evaluate the impact of TSA on myotube formation, HSMMs were allowed to differentiate in low-serum medium without TSA treatment, followed by a well-recognized protocol.^19,26^ MHC staining showed that TSA at 50 nM significantly enhanced myotube fusion when compared to 25 nM TSA-treated groups and the untreated control, as indicated by hypernucleated myotubes with increased cell size (Figs. 1e, f and Supplementary Fig. 1a). To detect the gene expression changes during myogenesis, through qPCR testing, we found that TSA at 50 nM significantly enhanced the expression of *Myod*, *Myf5*, *Myog*, *Myh2* and *Mrf4* genes (Fig. 1g). Furthermore, to rule out the possibility that the formation of larger, hypernucleated myotubes was due to an increased number of undifferentiated myoblasts available for fusion, a dose-dependent effect on myoblast proliferation activity was observed following TSA treatment. Compared to untreated cells, we did not find HDAC inhibition stimulate cell proliferation (Supplementary Fig. 1b). Taken together, these results indicated that HDAC inhibition with TSA administration on HSMMs promotes the formation of myotubes with increased cell size and enhanced myogenic gene expression. Based on the above results, it was also concluded that 50 nM TSA treatment could be used for subsequent experiments.

EVs, as nanosized facilitators of intercellular and intertissue communication, have been shown to mediate the paracrine effects in skeletal muscle.^27–29^ Here, EVs were isolated from TSA-treated (TSA-EVs) and untreated (UN-EVs) myoblast differentiation conditional medium via multiple centrifugation steps, ultracentrifugation, and density gradient separation. To minimize the likelihood of residual TSA in the final isolated EV preparation, we took care to thoroughly wash and pellet the EVs, discarding the supernatant. Transmission electron microscopy (TEM) imaging confirmed the presence of nanoparticles in both groups, exhibiting the characteristic size and spherical shape associated with EVs (Fig. 1h). Nanoparticle Tracking Analysis (NTA) demonstrated that those EVs exhibited a similar average in diameter (Fig. 1i). Besides, EVs from TSA-treated HSMM cultures exhibited a higher zeta potential compared to the UN-EV group, indicating altered surface properties of the EVs (Fig. 1j). We also found increased RNA quantity in TSA-EV when compared to the UN-EV group (Fig. 1k). Immunoblotting analysis verified the presence of CD9, CD81, and Alix proteins in both groups of EVs (Fig. 1l). Moreover, we also examined the internalization of these EVs by the hBMSCs. After 24 h incubation, we observed that TSA-EVs were internalized more by the human bone marrow mesenchymal stem cells (hBMSCs), indicating that the properties of TSA-EVs could influence their internalization and subsequently cellular responses (Fig. 1m, n). These findings propose that HDAC inhibition substantially promotes HSMM fusion and myogenic gene expression, while EVs from TSA-treated HSMM cultures exhibit enhanced properties.

### Trichostatin A-EVs prevent ovariectomized-induced bone loss

**Fig. 2:**
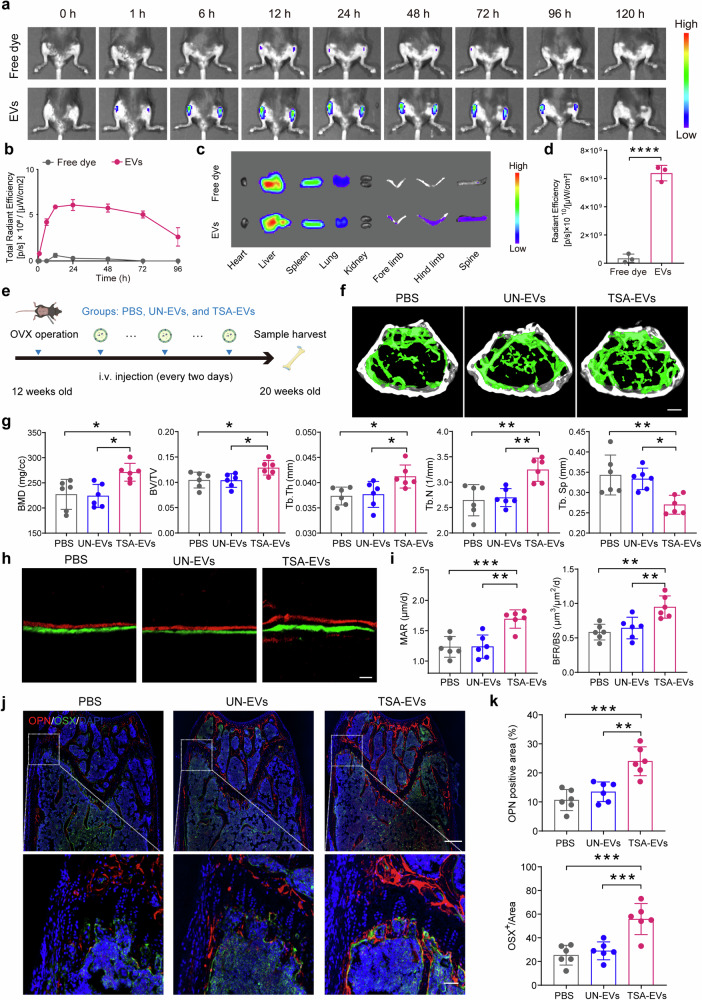
Trichostatin A (TSA)-EVs prevents ovariectomized (OVX)-induced bone loss. **a**, **b** Representative in vivo fluorescence images (**a**) and the quantification analysis (**b**) of the posterior limbs in 8-week C57BL/6J mice at the indicated time points after a single tail vein injection of free dye and EVs (*n* = 3 per group). **c** Representative fluorescence imaging of different organs in 8-week C57BL/6J mice 24 h after a single tail vein injection of free dye and EVs (*n* = 3 per group). **d** Quantification analysis of fluorescence in bone tissues harvested from mice (*n* = 3 per group). **e** Scheme showing that female C57BL/6 mice at 12-week-old received ovariectomy and then consecutive intravenous injections every two days with PBS, UN-EVs, and TSA-EVs for a total of 8 weeks. After treatment, mice were sacrificed, and femurs were harvested for further evaluation. **f** Representative micro-CT images of trabecular bone in the femur in 20-week C57BL/6J mice. Scale bar: 400 μm. **g** Micro-CT qualifications of BMD, BV/TV, Tb.Th, Tb.N, and Tb.Sp (*n* = 6 per group). **h**–**i** Representative images (**h**) of new bone formation at distal femur metaphysis assessed by bone histomorphometry analysis (**i**) of the differences in MAR and BFR/BS at distal femur across the three groups (*n* = 6 per group). Scale bar: 25 μm. **j**, **k** Representative images (**j**) of OSX (green) and OPN (red) immunostainings and quantification (**k**) of OSX^+^ and OPN^+^ area on distal femurs (*n* = 6 per group). Scale bar: 300 μm and 50 μm, respectively. All data are presented as the mean ± SD. *p < 0.05; **p < 0.01; ***p < 0.001; ****p < 0.0001. Statistical significance was determined by one-way ANOVA test

Fundamental evidence to support that EVs might mediate crosstalk between muscles and bones would show that the uptake of muscle-derived EVs is possible in the bone tissue. Therefore, we isolated EVs from differentiated HSMMs and labeled them with DIR, a red fluorescent dye that can label the lipid bilayers of EVs. Next, we intravenously injected 100 µg of these DIR-labeled EVs into 8-week-old mice, which were analyzed by intravital imaging, and free dye group without EVs were used as a negative control. Consistent with prior research, time-lapse imaging analysis showed weak or no fluorescence in the hind legs of mice treated with control-free dye. In contrast, there was a gradual increase in fluorescent signaling in the EV group from 0 to 24 h, though the signal gradually decreased thereafter (Fig. 2a, b). To further examine the distribution of EVs, major organs including the heart, liver, spleen, lungs, kidneys, anterior limbs, posterior limbs, and spine were harvested 24 h post-injection for imaging (Fig. 2c). The EV group exhibited stronger signal intensity in bone tissues, such as the limbs and spine, compared to the free dye control (Fig. 2d). Taken together, these findings indicated the biodistribution and accumulation of HSMM-EVs in bone tissues, which provide fundamental evidence supporting muscle mediated-EV targets on bones.

To investigate the pro-osteogenic effects of TSA-EVs, EVs from different culture conditions and PBS, were purified and systemically injected into 12-week-old ovariectomized (OVX) mice every two days for eight consecutive weeks (100 μg per dose per mouse) (Fig. 2e). Micro-CT analysis demonstrated that TSA-EVs significantly increased bone mineral density (BMD), bone volume fraction (BV/TV), trabecular thickness (Tb.Th), and trabecular number (Tb.N), while reducing trabecular separation (Tb.Sp) compared to the other groups (Fig. 2f, g). To evaluate bone quality, we performed mechanical stress testing, which revealed that the maximum load, bone stiffness, breaking energy, and energy to ultimate load were increased after TSA-EV injection (Supplementary Fig. 2). Additionally, bone histomorphometry was conducted to assess changes in mineral apposition rate (MAR) and bone formation rate over bone surface (BFR/BS). These parameters were significantly higher in mice receiving TSA-EV injections compared to those receiving UN-EV injections (Fig. 2h, i). Immunofluorescence staining of the distal femur further revealed increased expression of OSX and OPN in mice treated with TSA-EVs compared to those treated with UN-EVs (Fig. 2j, k). Taken together, these results demonstrated that TSA-EVs effectively promoted osteogenesis in vivo and potentially counteract bone loss.

### TSA-EVs promote osteogenic differentiation of hBMSCs and inhibit osteoclast formation

**Fig. 3:**
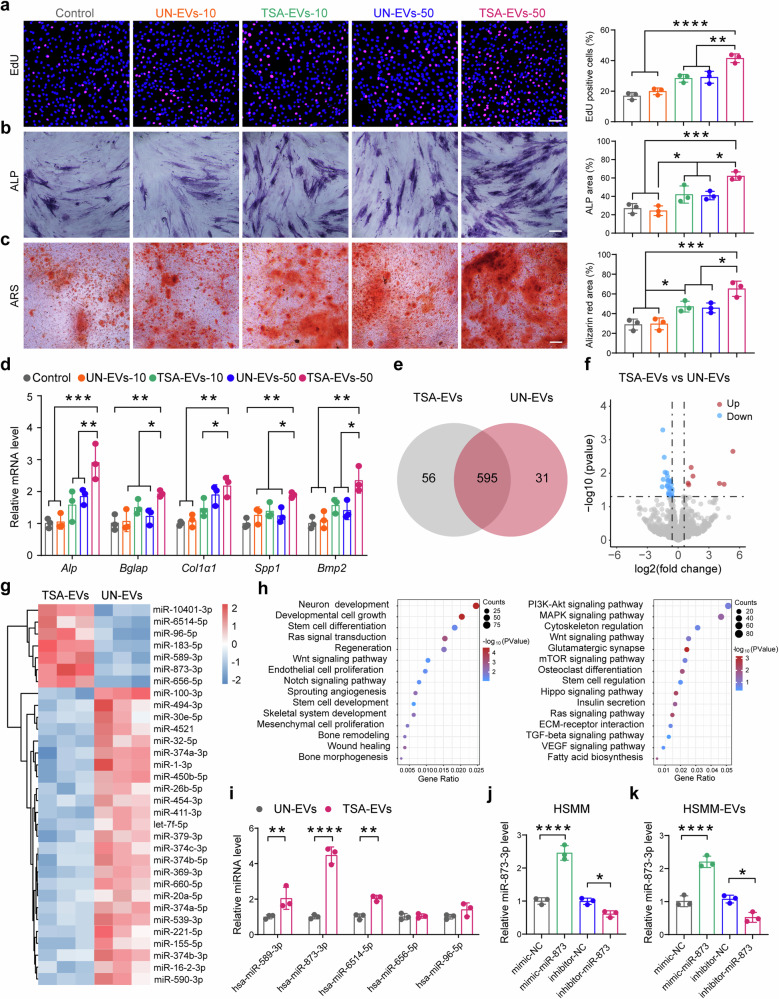
TSA-induced hyperacetylation promotes hBMSC activity and alters the microRNA profile of HSMM-derived EVs. **a** Cell proliferation of hBMSCs assessed by EdU staining. Scale bar: 100 μm (*n* = 3 per group). **b** Representative images of ALP staining of hBMSCs (left) and the corresponding quantitative analysis (right). Scale bar: 150 μm (*n* = 3 per group). **c** Representative images of ARS staining of hBMSCs (left) and the corresponding quantitative analysis (right). Scale bar: 150μm (*n* = 3 per group). **d** qPCR analysis of osteogenic markers *Alp*, *Bglap*, *Col1a1*, *Spp1*, and *Bmp2* after 14 days of osteogenic induction (*n* = 3 per group). **e** Venn diagram comparing microRNAs differentially expressed from TSA-EVs and UN-EVs. **f** Volcano plot showing the differentially expressed miRNAs from TSA-EVs and UN-EVs. **g** Hierarchical clustering analysis of microRNAs that were differentially expressed between TSA-EVs and UN-EVs (*n* = 3 per group). **h** Differentially expressed miRNAs were subjected to gene ontology (GO) and Kyoto Encyclopedia of Genes and Genomes (KEGG) pathways analysis. **i** Validation of the top five elevated miRNAs including hsa-miR-589-3p, hsa-miR-873-3p, hsa-miR-6514-5p, hsa-miR-656-5p and hsa-miR-96-5p between TSA-EVs and UN-EVs using qPCR (*n* = 3 per group). **j** The relative expression level of miR-873 in myoblasts transfected with miR-873 mimics, miR-873 inhibitor, or their negative control group (*n* = 3 per group). **k** The relative expression level of miR-873 in EVs derived from myoblasts transfected with miR-873 mimics, miR-873 inhibitor, or their negative control (*n* = 3 per group). qPCR results are presented as fold change relative to control. All data are presented as the mean ± SD. *p < 0.05; **p < 0.01; ***p < 0.001; ****p < 0.0001. Statistical significance was determined by one-way ANOVA test

To further confirm the osteogenic effects of TSA-EVs, we evaluated the proliferation of hBMSCs and induced osteogenic differentiation using UN-EVs and TSA-EVs at concentrations of 10 μg/ml (UN-EV-10 and TSA-EV-10) and 50 μg/ml (UN-EV-50 and TSA-EV-50), respectively. TSA-EV treatment significantly enhanced hBMSCs proliferation in a dose-dependent manner, as demonstrated by EdU assays and live cell imaging, compared to cells treated with UN-EVs or left untreated (Fig. 3a and Supplementary Fig. 3). The impact of TSA-EVs on hBMSCs extracellular matrix mineralization was further assessed by measuring alkaline phosphatase (ALP) activity and calcium deposition. Throughout 14 days of osteogenic differentiation, the TSA-EV group exhibited a higher intensity of ALP staining compared to the control and UN-EV groups (Fig. 3b). By day 21 of differentiation, the TSA-EV group demonstrated increased alizarin red S (ARS) staining intensity, with prominent calcium nodule formation, compared to the control and UN-EV groups (Fig. 3c). Moreover, treatment with 50 μg/ml of UN-EVs (UN-EV-50) and TSA-EVs (TSA-EV-50) further enhanced calcium deposition in hBMSCs compared to cells treated with 10 μg/ml EVs (UN-EV-10 and TSA-EV-10). Notably, the TSA-EV-50 group showed a significantly greater accumulation of calcium deposits and an increased number of mineralized nodules compared to the UN-EV-50 treated cells. We also assessed the expression of osteogenesis-related genes, finding that after 14 days of differentiation, the expression levels of *Alp*, *Bglap*, *Col1a1*, *Spp1*, and *Bmp2* were markedly upregulated in the TSA-EV group compared to the UN-EV and control groups (Fig. 3d). Collectively, these results highlight the potential of TSA-EVs to enhance hBMSC proliferation and osteogenic differentiation, leading to increased extracellular matrix mineralization.

To investigate the effects of skeletal muscle-derived EVs on osteoclast formation, we performed TRAP staining to assess osteoclast differentiation using EVs derived from untreated HSMMs (UN-EVs) and TSA-treated HSMMs (TSA-EVs). As shown in the TRAP staining images and quantitative analysis of TRAP-positive cell area, TSA-EVs (50 μg/ml) significantly inhibited osteoclast formation compared to the UN-EVs and control groups (Supplementary Fig. 4a). Specifically, the TRAP-positive cell area was markedly reduced in the TSA-EVs groups, with the highest inhibition observed at the 50 μg/ml concentration (Supplementary Fig. 4b). In contrast, the UN-EVs groups did not show a significant inhibitory effect on osteoclast formation compared to the control. Furthermore, we assessed the expression of osteoclast differentiation-related genes, including Cathepsin K (*Ctsk*), DC-STAMP, and Acid Phosphatase 5 (*Acp5*), through qPCR analysis (Supplementary Fig. 4c). Our data revealed that the expression levels of these genes were significantly lower in the TSA-EVs groups compared to the UN-EVs and control groups, further supporting the inhibitory effect of TSA-EVs on osteoclast differentiation. These results indicate that skeletal muscle-derived EVs, particularly those derived from TSA-treated HSMMs, have a significant inhibitory effect on osteoclast formation.

By demonstrating both the promotion of osteogenic differentiation of hBMSCs and the inhibition of osteoclast formation, our findings highlight the dual role of TSA-EVs in regulating bone remodeling. This underscores the potential of TSA-EVs as a promising therapeutic approach for enhancing bone regeneration and maintaining bone health.

### TSA-induced hyperacetylation alters the microRNA profile of HSMM-derived EVs

EVs secreted by various cell types are composed of a lipid bilayer that encases a range of biomolecules, including proteins, lipids, and nucleic acids such as miRNAs.^30–32^ To further investigate the impact of epigenetic modifications on HSMM-derived EVs and their potential role in enhancing osteoinductive properties, we conducted a microRNA expression profiling of these EVs. The Venn diagram incorporated into our research offers a visual representation of the contrasting microRNA expression patterns between TSA-EVs and UN-EVs. Notably, it underscores the co-occurrence of 595 microRNA species shared between both cohorts, alongside 56 microRNA species uniquely expressed in TSA-EVs and 31 microRNA species specific to UN-EVs (Fig. 3e). Volcano plots highlighted the differences in microRNA expression between TSA-EVs and UN-EVs (Fig. 3f), with the differentially expressed microRNAs listed in Supplementary Table 1. Hierarchical clustering further confirmed the distinct microRNA expression profiles in TSA-EVs compared to UN-EVs, and the top 7 upregulated miRNAs identified in TSA-EVs were hsa-miR-10401-3p, hsa-miR-6514-5p, hsa-miR-96-5p, hsa-miR-183-5p, hsa-miR-589-3p, hsa-miR-873-3p, and hsa-miR-656-5p (Fig. 3g). To explore the functions of the differentially expressed miRNAs, we conducted Gene Ontology (GO) and Kyoto Encyclopedia of Genes and Genomes (KEGG) analyses of their target genes. The GO analysis revealed enrichment in terms related to osteogenesis, including developmental cell growth, regeneration, positive regulation of canonical Wnt signaling pathway, regulation of Notch signaling pathway, stem cell development, cranial skeletal system development, and mesenchymal cell proliferation. KEGG analysis also identified several signaling pathways associated with osteoblast differentiation, including the PI3K-Akt signaling pathway, MAPK signaling pathway, mTOR signaling pathway, and Wnt signaling pathway (Fig. 3h).

Based on this miRNA profiling data, next, we selected the top 5 upregulated miRNAs for further validation using qPCR. We found that three miRNAs, including hsa-miR-589-3p, hsa-miR-873-3p, and hsa-miR-6514-5p were significantly upregulated in TSA-EVs compared to UN-EVs, with hsa-miR-873-3p demonstrating the highest expression fold change (Fig. 3i). To determine whether TSA is increasing expression or packaging of miRNAs into EVs, we also measured miRNA expression using qPCR in vehicle and TSA-treated HSMMs. We found that miR-589-3p, miR-873-3p, miR-6514-5p, miR-656-5p, and miR-96-5p are significantly upregulated in TSA-treated HSMMs compared to the vehicle-treated cells, while no significant changes were observed for miR-183-5p and miR-10401-3p are observed between these two groups (Supplementary Fig. 5). These results indicated that TSA treatment enhances the expression of specific miRNAs, which are then incorporated into EVs. While miR-873 has been found to have either tumor-suppressive^33^ or tumor-promoting effects^34^ in different types of cancer, its expression was upregulated upon parathyroid hormone treatment in rat osteoblasts and downregulated HDAC4 protein expression, thereby stimulating *MMP-13* expression in these cells.^35^ Building on our miRNA expression findings and previous research, we focused on hsa-miR-873-3p to determine whether TSA-EVs promote osteogenesis through the transfer of hsa-miR-873-3p. To gain mechanistic insights into the role of EV-derived hsa-miR-873-3p in TSA-EV-induced osteogenesis, we transfected HSMMs with miR-873 mimics, miR-873 inhibitors, or their respective negative controls. The efficiency of transfection was confirmed using qPCR (Fig. 3j). Next, we isolated EVs from the above groups, respectively, and detected the miR-873 expression. We found that the miR-873 mimics caused an increase in the expression of miR-873, while the miR-873 inhibitor led to a decrease in miR-873 expression in EVs compared to their negative control (Fig. 3k). These data support the notion that TSA-EVs promote osteogenesis, and miR-873-3p is upregulated in TSA-treated HSMMs and their EVs.

### TSA-EV-shuttled miR-873-3p from muscle promotes bone regeneration

**Fig. 4:**
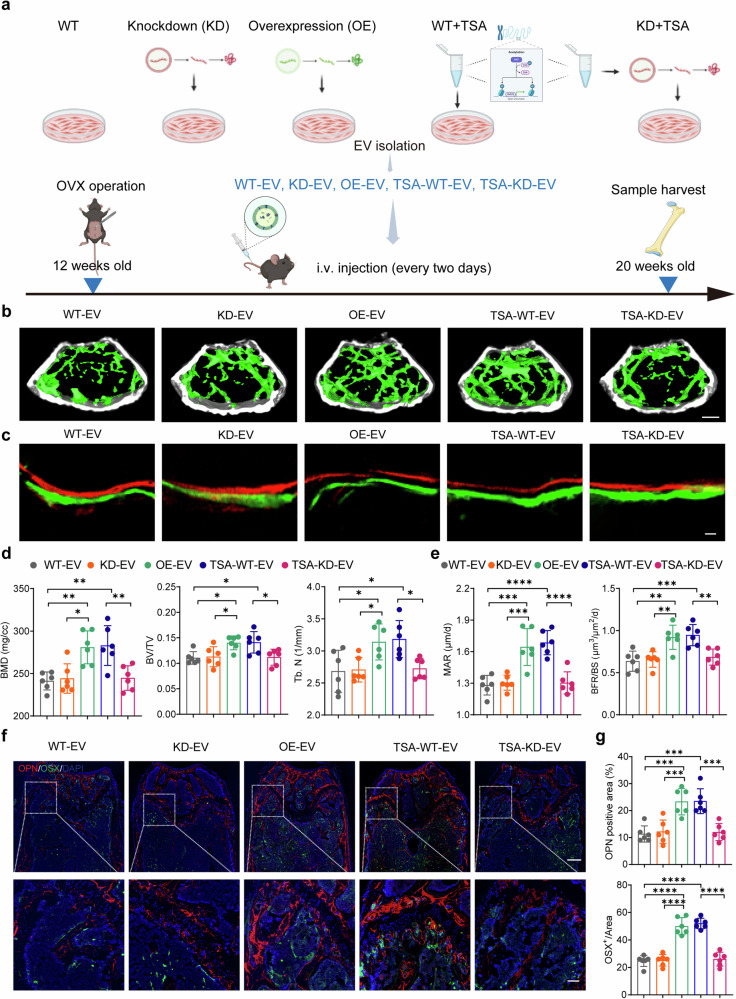
TSA-EV-shuttled miR-873-3p from muscle promotes bone regeneration. **a** The female C57BL/6 mice at 12-week-old received ovariectomy and then consecutive intravenous injections every two days with EVs purified from untreated WT HSMMs (WT-EV), miR-873-3p knockdown HSMMs (KD-EV), miR-873-3p overexpressing HSMMs (OE-EV), TSA-treated WT HSMMs (TSA-WT-EV) and TSA-treated miR-873-3p knockdown HSMMs (TSA-KD-EV) for a total of 8 weeks. After treatment, mice were sacrificed, and femurs were harvested for further evaluation. **b** Representative micro-CT images of trabecular bone in the femur in 20-week C57BL/6J mice. Scale bar: 400 μm. **c** Representative images of new bone formation at distal femur metaphysis assessed by bone histomorphometry analysis. Scale bar: 25 μm. **d** Qualifications of BMD, BV/TV and Tb.N after indicated treatments using the micro-CT images in **b** (*n* = 6 per group). **e** Quantitative analysis of BFR/BS and MAR in (**c**) (*n* = 6 per group). **f**, **g** Representative images (**f**) of OSX (green) and OPN (red) immunostainings and quantifications (**g**) on distal femurs (*n* = 6 per group). Scale bar: 300 μm and 50 μm, respectively. All data are presented as the mean ± SD. *p < 0.05; **p < 0.01; ***p < 0.001; ****p < 0.0001. Statistical significance was determined by one-way ANOVA test

Recognizing the potential of TSA-EVs to enhance bone regeneration, and the key role played by upregulated miR-873-3p, we endeavored to understand if EVs carrying an excess of miR-873-3p could facilitate beneficial effects, while the EVs lacking this miRNA could adversely affect bone formation, and crucially, whether TSA treatment itself contributes significantly to these observed effects. To test this possibility, we intravenously injected EVs purified from untreated WT HSMMs (WT-EV), miR-873-3p knockdown HSMMs (KD-EV), miR-873-3p overexpressing HSMMs (OE-EV), TSA-treated WT HSMMs (TSA-WT-EV) and TSA-treated miR-873-3p knockdown HSMMs (TSA-KD-EV) into 12-week-old OVX mice every two days for eight consecutive weeks (100 μg per dose per mouse) (Fig. 4a). After the treatment, micro-CT analysis of the distal femur revealed significant differences in trabecular architecture among the groups, and mice treated with OE-EV and TSA-WT-EV exhibited significant increases in bone mass and well organized trabecular architecture (Fig. 4b). Micro-CT analysis revealed that OE-EV and TSA-WT-EV significantly increased BMD, BV/TV and Tb. N compared to other groups (Fig. 4d). Specifically, the TSA-KD-EV group showed reduced bone mass compared to the TSA-WT-EV group due to miR-873 knockdown, indicating that even after TSA treatment, the knockdown of miR-873 still affects bone formation.

Further, the results of bone histomorphometry analysis showed that OE-EV and TSA-WT-EV treatment led to improved new bone formation, as evidenced by increased MAR and BFR/BS values. In contrast, The KD-EV and TSA-KD-EV groups showed significantly lower MAR and BFR/BS, consistent with reduced bone formation activity (Fig. 4c, e). Immunofluorescence staining of the distal femur also showed that the number of OSX^+^ and OPN^+^ cells was increased in mice treated with TSA-WT-EV and OE-EV, while diminished in the KD-EV and TSA-KD-EV groups (Fig. 4f, g). Taken together, these data indicate that TSA-EV-shuttled miR-873-3p is essential for bone formation, as high expression of miR-873-3p increases bone mass and quality, while its knockdown abolishes these effects. Furthermore, the lack of significant bone regeneration in the TSA-KD-EV group compared to the TSA-WT-EV group demonstrates that even TSA treatment cannot compensate for the loss of miR-873-3p, highlighting the essential and non-redundant role of miR-873-3p in promoting bone health.

### TSA-HSMM-EVs mimic the effects of exercise on bone formation

**Fig. 5:**
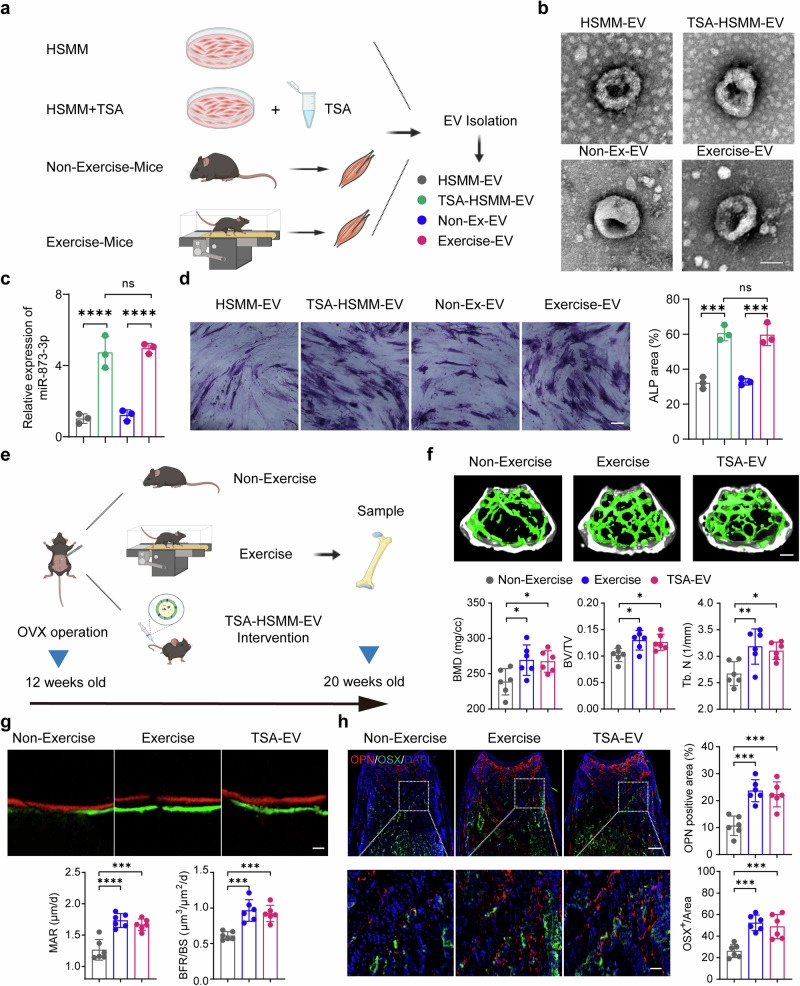
TSA-HSMM-EVs mimic the effects of exercise on bone formation. **a** Schematic representation of the experimental design. EVs were isolated from HSMMs, TSA-treated HSMMs, non-exercising OVX mice, and exercising OVX mice. EVs from each source were characterized and their effects on osteogenic differentiation and bone formation were evaluated. **b** Transmission electron microscopy (TEM) images of EVs isolated from HSMMs (HSMM-EV), TSA-treated HSMMs (TSA-HSMM-EV), non-exercising OVX mice (Non-Ex-EV), and exercising OVX mice (Exercise-EV). Scale bar: 50 nm. **c** Relative expression of miR-873-3p in EVs derived from different sources, as measured by qPCR (*n* = 3 per group). **d** ALP staining of hBMSCs treated with EVs from the different sources. Quantification of ALP-positive areas is shown on the right. Scale bar : 150 µm (*n* = 3 per group). **e** Schematic of the in vivo experimental setup. OVX mice were subjected to non-exercise, exercise, or TSA-HSMM-EV treatment, and bone samples were harvested at 20 weeks old. **f** Representative micro-CT images of the distal femur and quantification of BMD, BV/TV, and Tb.N in the three groups. Scale bar: 400 μm. (*n* = 6 per group). **g** Bone histomorphometry showing MAR and BFR/BS in the three groups. Scale bar: 25 µm (*n* = 6 per group). **h** Immunofluorescence staining for OPN and OSX in the distal femur. Quantification of OPN-positive and OSX-positive areas is shown on the right. Scale bar: 300 μm and 50 μm, respectively. (*n* = 6 per group). All data are presented as the mean ± SD. *p < 0.05; **p < 0.01; ***p < 0.001; ****p < 0.0001. Statistical significance was determined by one-way ANOVA test

Physical exercise is well-known for its positive effects on bone health, primarily through mechanical loading and the release of bioactive molecules from skeletal muscle.^36^ To investigate whether TSA treatment of HSMMs mimics the effects of exercise on EV content and function, we designed an experiment with four groups (Fig. 5a): HSMM-EV (EVs from untreated HSMMs), TSA-HSMM-EV (EVs from TSA-treated HSMMs), Non-Exercise-EV (EVs from non-exercised OVX mice muscle), and Exercise-EV (EVs from exercised OVX mice muscle). EVs were isolated from the muscle tissues and conditioned media of the HSMMs, followed by characterization using transmission electron microscopy (TEM), confirming consistent EV morphology across the groups (Fig. 5b). We also measured the expression level of miR-873-3p in the isolated EVs using qPCR. The results showed significantly higher levels of miR-873-3p in both TSA-HSMM-EV and Exercise-EV groups compared to the HSMM-EV and Non-Exercise-EV groups, indicating that both TSA treatment and exercise upregulate miR-873-3p in EVs (Fig. 5c). To evaluate the osteogenic potential of these EVs, we treated hBMSCs with each group of EVs (50 μg/ml) and performed ALP staining and quantification. Both TSA-HSMM-EV and Exercise-EV groups showed significantly increased ALP activity compared to HSMM-EV and Non-Exercise-EV groups (Fig. 5d).

To further test the bone formation efficacy of these EVs in vivo (100 μg per dose per mouse), we used OVX mice divided into three groups: Non-Exercise (control), Exercise, and TSA-HSMM-EV treatment (Fig. 5e). Micro-CT analysis of the distal femur showed significant improvements in bone parameters of BMD, BV/TV, and Tb. N in both the Exercise and TSA-HSMM-EV groups compared to the Non-Exercise group (Fig. 5f). Bone histomorphometry supported these findings, with higher MAR and BFR/BS observed in the Exercise and TSA-HSMM-EV groups (Fig. 5g). Immunofluorescence staining for osteogenic markers OPN and OSX also revealed increased positive areas in these two groups (Fig. 5h). These results demonstrate that TSA-HSMM-EVs can effectively mimic the beneficial effects of exercise on bone formation, achieving comparable outcomes in terms of miR-873-3p expression and osteogenic potential. This supports the notion that HDAC inhibition via TSA treatment can reproduce some aspects of the exercise response, particularly through the upregulation of miR-873-3p in EVs. Consequently, TSA-EVs present a viable alternative for enhancing bone health in individuals who are unable to engage in physical exercise.

### Extracellular vesicle transfers HSMM-miR-873-3p to hBMSCs for dictating osteogenesis

**Fig. 6:**
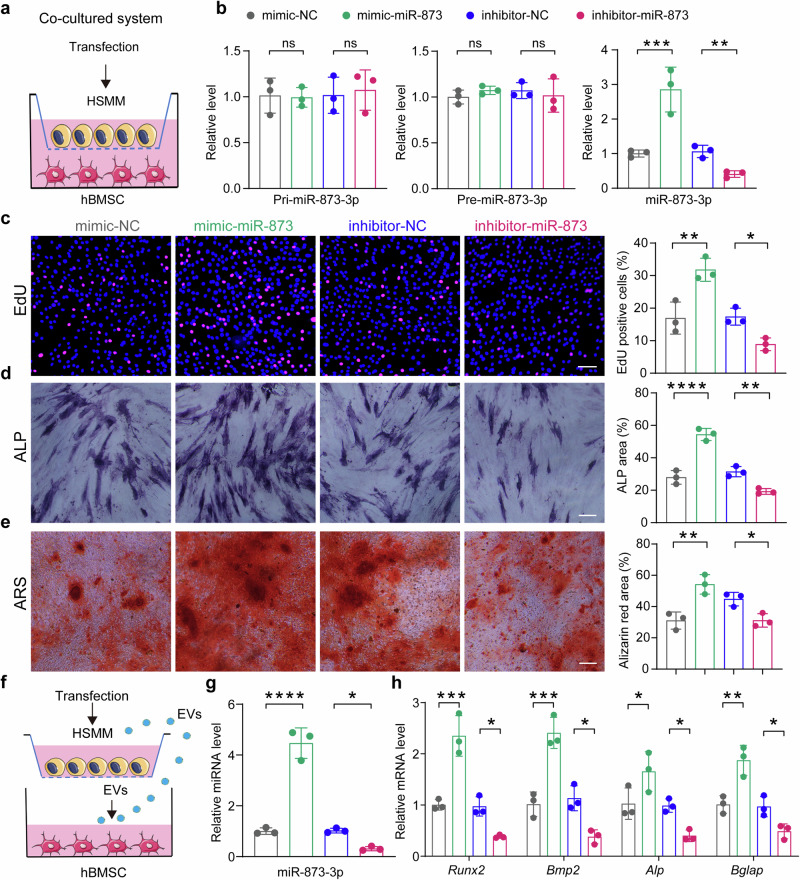
Extracellular vesicle transfers HSMM-miR-873-3p to hBMSCs for dictating osteogenesis. **a** Schematic representation illustrating the design of the co-culture experiments. The hBMSCs were co-cultured with the HSMMs transfected with miR-873 mimics, miR-873 inhibitor, or their negative control group. **b** The relative expression analysis of pri-miR-873, pre-miR-873, and miR-873 in hBMSCs after co-culture with HSMMs for 24 h using qPCR (*n* = 3 per group). **c** Cell proliferation analysis of hBMSCs co-culture with HSMMs transfected with miR-873 mimics, miR-873 inhibitor, or their negative control group. Scale bar: 100μm (*n* = 3 per group) at 24 h after co-culture. **d** Representative images of ALP staining of hBMSCs (left) and the corresponding quantitative analysis (right). Scale bar: 150 μm (*n* = 3 per group). **e** Representative images of ARS staining of hBMSCs (left) and the corresponding quantitative analysis (right). Scale bar: 150 μm (*n* = 3 per group). **f**, **g** Schematic representation (**f**) and analysis (**g**) of miR-873 expression levels in hBMSCs after treated by different transfected HSMMs’ EVs at 50 μg/ml for 24 h (*n* = 3 per group). **h** qPCR analysis of osteogenic markers *Runx2*, *Bmp2*, *Alp* and *Bglap* in hBMSCs after co-culture with HSMMs at 48 h after co-culture (*n* = 3 per group). qPCR results are presented as fold change relative to control. All data are presented as the mean ± SD. *p < 0.05; **p < 0.01; ***p < 0.001, ****p < 0.0001. Statistical significance was determined by one-way ANOVA test

Having demonstrated that HSMM-derived miR-873-3p could regulate bone regeneration in vivo, we sought to investigate whether it can be transferred to hBMSCs, and serves as a biological messenger to regulate osteogenic differentiation. To accomplish this, we co-cultured HSMMs with hBMSCs in a Transwell system using a 0.4 μm pore polyethylene terephthalate (PET) membrane, as previously described.^37^ The HSMMs were transfected with miR-873 mimics, miR-873 inhibitor, or their negative control, and then incubated with hBMSCs, respectively (Fig. 6a). After 24 h, qPCR testing of hBMSCs confirmed that the expression of miR-873-3p was significantly increased after incubation with miR-873 mimics-transfected HSMMs, and decreased with miR-873 inhibitor group. However, no significant difference in the levels of pri-miR-873-3p or pre-miR-873-3p in hBMSCs was observed between these co-cultures (Fig. 6b). Next, we further assessed the biological activity of hBMSCs during co-culture with different HSMM groups. EdU assays showed that hBMSCs incubated with miR-873 mimics and miR-873 inhibitor-transfected HSMMs exhibited an increase and inhibition in proliferation rate at 24 h after co-culture, respectively (Fig. 6c). The effects of HSMM-miR-873 on hBMSCs extracellular matrix mineralization were assessed by quantifying ALP activity and calcium deposition. During 14 days and 21 days of osteogenic differentiation, the miR-873 mimics group showed an elevated intensity of ALP and ARS staining when compared to the negative control group, and miR-873 inhibitor group showed decreased intensity (Fig. 6d, e). These results suggest that the elevated miR-873-3p in HSMMs may contribute to the increased miR-873-3p levels in hBMSCs, thereby enhancing osteogenic activity in vitro. To further examine whether EVs were responsible for the miR-873 transfer and whether HSMM-EV-miR-873 transfer was capable of affecting hBMSC miR-21 in physiological status, we collected EVs from the different HSMMs groups and treated hBMSCs, respectively (Fig. 6f). We found that while the pri-miR-873 and pre-miR-873 levels in hBMSCs were not influenced (Supplementary Fig. 7), miR-873-overxpressed HSMM EVs increased miR-873 expression in hBMSCs, and that EVs from miR-873-underexpressed HSMMs had diminished effects (Fig. 6g). Moreover, we assessed the mRNA expression of osteoblast activity-related marker genes (*Runx2*, *Bmp2*, *Alp*, and *Bglap*) in hBMSCs after after co-culture with different HSMM-EVs at 48 h after co-culture. The mRNA levels of those genes were consistent with the changes of miR-873 levels (Fig. 6h). Altogether, these data indicated that HSMM-derived miR-873-3p was transferred through EVs to hBMSCs and promoted osteogenesis process.

Since we also found that other miRNAs upregulated in TSA-EVs, specifically miR-589-3p and miR-6514-5p, we further aimed to figure our their effects on osteogenesis. hBMSCs were then transfected with miR-589-3p mimics, miR-6514-5p mimics, and corresponding negative controls (mimic-NC). The ALP and ARS staining results showed no significant differences in osteogenic activity between the miRNA mimic groups and their respective controls (Supplementary Fig. 6a, b, d, e). Additionally, qPCR analysis showed no significant differences in the expression levels of the osteogenic markers *Runx2*, *Bmp2*, *Alp*, and *Bglap* between the mimic and control groups (Supplementary Fig. 6c, f). These findings suggest that while miR-589-3p and miR-6514-5p are upregulated in TSA-EVs, they do not significantly impact the osteogenic differentiation of hBMSCs. Therefore, the beneficial effects of TSA-EVs on bone formation are primarily driven by miR-873-3p.

### Hsa-miR-873-3p promotes the process of osteogenesis via targeting *CNN2*

**Fig. 7:**
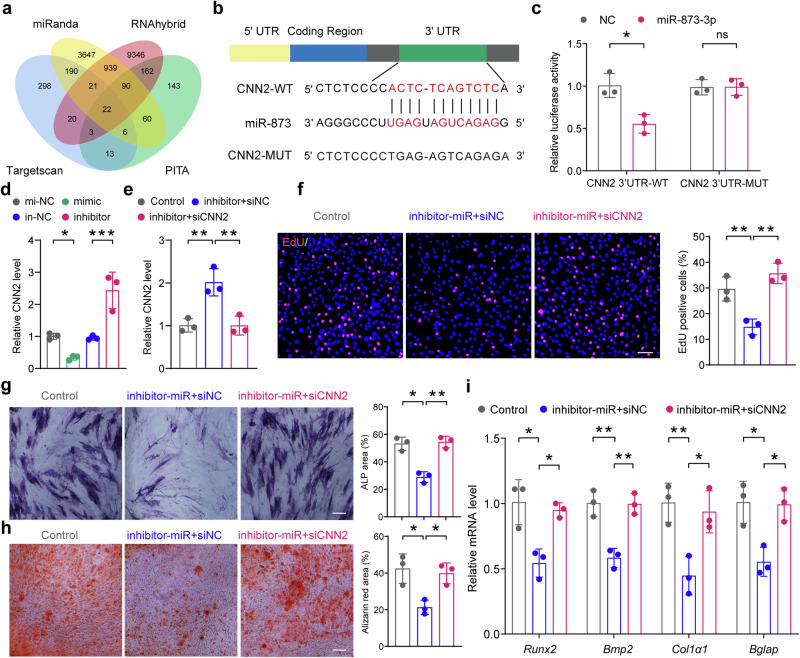
Has-miR-873-3p regulates the process of osteogenesis via targeting *CNN2*. **a** Venn diagram showing miR-873 targets identified by four different independent microRNA-target-predicting programs (Targetscan, miRanda, RNAhybrid, and PITA). **b** The predicted miR-873 targeting sequence in the 3′-UTR of CNN2. **c** Luciferase reporter assay was performed to confirm that CNN2 is the target gene of miR-873 (*n* = 3 per group). **d** The relative expression analysis of *CNN2* in hBMSCs after co-culture with HSMMs for 24 h using qPCR (*n* = 3 per group). **e** The relative expression of *CNN2* in hBMSCs after transfection with miR-873 inhibitor and si*CNN2* or siNC by qPCR (*n* = 3 per group). **f** Cell proliferation analysis of hBMSCs transfected with miR-873 inhibitor and si*CNN2* or siNC by EdU staining. Scale bar: 100 μm (*n* = 3 per group). **g** Representative images of ALP staining of hBMSCs (left) and the corresponding quantitative analysis (right). Scale bar: 150 μm (*n* = 3 per group). **h** Representative images of ARS staining of hBMSCs (left) and the corresponding quantitative analysis (right). Scale bar: 150 μm (*n* = 3 per group). **i** qPCR analysis of osteogenic markers *Runx2*, *Bmp2*, *Col1a1*, and *Bglap* after 14 days of osteogenic induction (*n* = 3 per group). qPCR results are presented as fold change relative to control. All data are presented as the mean ± SD. *p < 0.05; **p < 0.01; ***p < 0.001. Statistical significance was determined by one-way ANOVA test

To explore the potential mechanism by which TSA-EV-shuttled miR-873-3p promotes hBMSC activity, we independently used four online databases—TargetScan, miRanda, RNAhybrid, and PITA—to identify predicted mRNA targets for miR-873-3p (Fig. 7a). Additionally, we constructed a miRNA-mRNA interaction network using Cytoscape software (Supplementary Fig. 8). Based on these results, H2 calponin (*CNN2*), an actin-binding protein involved in cytoskeletal organization,^38,39^ was predicted as a potential target gene of miR-873-3p. Our selection of CNN2 as the target gene of interest was based on its crucial role in regulating the cytoskeleton and cellular contractility. CNN2 is particularly significant due to its widespread expression in muscle and none-muscle tissues.^40,41^ CNN2 regulates cytoskeletal organization and cell motility, such as cell proliferation, differentiation, and migration, which are crucial for osteogenesis and bone regeneration.^42^ Moreover, the expression of CNN2 in tissues undergoing high mechanical tension and its regulatory role in osteoblast activity underscore its importance in bone metabolism. Dysregulation of CNN2 has been implicated in various diseases, including cancer and inflammatory conditions, and it negatively regulates osteoblast activity.^40,43^ Targeting CNN2 thus provides a strategic avenue to enhance bone formation. To validate CNN2 3’ UTR as a direct target of miR-873, we performed a luciferase reporter assay in transfected HEK 293t cells (Fig. 7b). Co-transfection with the downregulated miR-873 and the CNN2 WT luciferase construct resulted in increased luciferase activity, an effect not observed with the MUT construct (Fig. 7c). Moreover, the qPCR analysis demonstrated decreased *CNN2* expression and increased *CNN2* expression in hBMSCs after incubation with miR-873 mimics and miR-873 inhibitor-transfected HSMMs, respectively (Fig. 7d). Given calponin’s role as a negative regulator of osteoblast functions,^44,45^ and supported by our gene expression and luciferase assay data, we confirmed CNN2 as a target of miR-873.

**Fig. 8:**
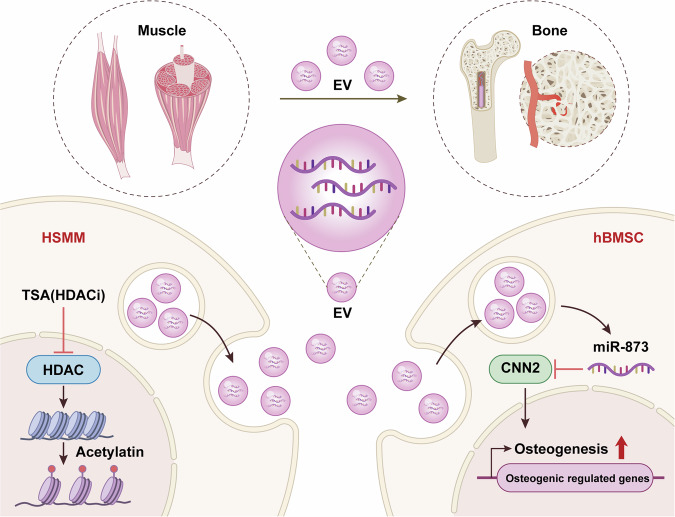
**Conceptual diagram demonstrating the generation and subsequent transfer of EVs from HDAC-inhibited HSMMs to hBMSCs**. This non-exercising dependent transfer from muscle enhances bone regeneration, a process modulated by the interaction between has-miR-873-3p microRNA and H2 Calponin (*CNN2*). Histone acetylation could mediate HSMMs to produce EVs containing excessive miR-873-3p. These EV-derived miR-873-3p could be directly transferred to hBMSCs, and targetes *CNN2*. By degrading *CNN2* mRNA in hBMSCs, TSA-treated HSMM-EVs promote the process of osteogenesis, and enhance bone mass. The diagram was edited using Adobe Illustrator, Adobe Photoshop, and CINEMA 4D

To further investigate the relationship between EV-miR-873 and CNN2, as well as CNN2’s role in regulating hBMSC activity, we conducted a series of in vitro rescue experiments. We transfected miR-873 inhibitor with siNC or si*CNN2* into hBMSCs and detected expression levels by qPCR. Results demonstrated that hBMSCs transfected with miR-873 inhibitor and si*CNN2* showed significantly lower expression of *CNN2* when compared to those transfected with miR-873 inhibitor and siNC (Fig. 7e). In the EdU proliferation assays, we found that co-transfected with miR-873 inhibitor and si*CNN2* promoted cell proliferation when compared to miR-873 inhibitor and siNC co-transfection (Fig. 7f). ALP and ARS staining also revealed that inhibiting CNN2 counteracted the negative impact of the miR-873 inhibitor on the osteogenic capacity of hBMSCs (Fig. 7g, h). Further, the qPCR analysis of osteogenic expression, including *Runx2*, *Bmp2*, *Col1a1*, and *Bglap*, demonstrated that silencing of *CNN2* rescued the decreased osteogenesis capability caused by miR-873 inhibition (Fig. 7i). These data suggested that inhibiting *CNN2* in hBMSCs could abolish the negative role of miR-873-inhibitor on cell proliferation and osteogenesis. Taken together, these results demonstrated that histone acetylation could mediate HSMMs to produce EVs containing excessive miR-873-3p. These EV-derived miR-873-3p could be directly transferred to hBMSCs, and promoted the process of osteogenesis by silencing *CNN2* (Fig. 8).

## Discussion

It is well-documented that muscle decline often precedes bone loss, particularly in aging populations and individuals with certain clinical conditions such as sarcopenia and osteoporosis.^46^ This interrelationship between muscle and bone health is a key consideration in developing therapeutic strategies. Muscle tissue is essential for maintaining bone integrity through mechanical loading and the secretion of bioactive factors that regulate bone metabolism. Given this relationship, interventions targeting muscle health can have significant downstream effects on bone health. Our research uncovers a novel muscle-to-bone communication pathway, where skeletal muscle cells transmit genetic signals to bone cells. Our findings highlight that human myoblast-derived miR-873-3p, facilitated by histone hyperacetylation in HSMMs, plays a crucial role in bone formation. By degrading CNN2 mRNA in hBMSCs, miR-873-3p enhances bone mass, offering a new understanding of the muscle-bone interplay. Notably, EVs emerge as key vehicles for transporting miR-873-3p to bone sites, thereby mitigating bone loss. Conversely, reducing miR-873-3p expression correlates with decreased bone density. This study opens a new avenue in understanding the complex physiological regulation of muscle metabolism and lays the groundwork for developing osteoporosis treatments.

Muscle metabolism is influenced by various epigenetic mechanisms, including histone acetylation. Skeletal muscle adaptations to metabolic stressors like physical exercise, obesity, or high-fat diets (HFDs) are modulated through histone deacetylases (HDACs). For instance, exercise triggers Class II HDAC nuclear export in skeletal muscles, while obesity and HFDs engage both Class I and II HDACs.^47–49^ Besides, multiple preclinical studies have highlighted the potential therapeutic benefits of HDACi in regulating whole-body metabolism in rodent models of metabolic disorders. For example, the pan-HDACi sodium butyrate and TSA have demonstrated efficacy in improving insulin sensitivity and energy expenditure in HFD-fed mice,^50^ as well as increasing the liver expression and serum levels of FGF21.^51^ TSA, a naturally-derived HDACi, has been reported to accelerate osteogenic differentiation by inducing hyperacetylation, which leads to chromatin remodeling and activation of transcription factors.^52^ Chromatin remodeling enhances accessibility to osteoblast-related genes, while HDAC inhibition and the hyperacetylation of non-histone proteins boost the activity and stability of key osteogenic transcription factors, such as Runx2.^53^ In our study, TSA exposure was performed on myoblasts prior to their differentiation into myotubes to enhance the differentiation potential and therapeutic properties of these cells. HDAC inhibition at the myoblast stage primes these cells epigenetically, making them more responsive to differentiation signals and leading to more robust myotube formation. This approach is relevant because satellite cells (muscle stem cells) and myoblasts play a crucial role in muscle repair and regeneration, even in differentiated muscle tissue. Our study further enriches this domain by demonstrating how TSA enhances myotube fusion and muscle metabolism primarily through histone hyperacetylation. This process facilitates osteogenic differentiation and underscores the promising role of epigenetic modulation in bone health optimization, providing a foundation for innovative therapeutic strategies to mimic the benefits of exercise, particularly for patients unable to engage in physical activity.

The paracrine effects of skeletal muscle have been reported to be mediated by various secreted factors, including cytokines, growth factors, extracellular matrix proteins, lipid signaling molecules, and extracellular vesicles (EVs), which are important mediators in inter-tissue communication. HDAC inhibitors, such as Trichostatin A (TSA), are known to influence the secretion of these factors, thereby enhancing bone formation. Specifically, HDAC inhibitors can upregulate the secretion of growth factors like Bone Morphogenetic Proteins (BMPs), which are crucial for osteoblast differentiation and bone formation. Additionally, Insulin-like Growth Factor 1 (IGF-1), which promotes osteoblast proliferation and differentiation, is also enhanced by HDAC inhibitors.^54^ Researchers also discovered that HDAC inhibitor can induce the secretion of interferon-β, which plays a role in inhibiting the differentiation of osteoclasts.^55^ Cytokines such as tumor necrosis factor-alpha (TNF-α) also play significant roles in bone metabolism and is downregulated by HDAC inhibitors.^56^ Besides, phenylbutyrate and trichostatin A were shown to suppress the secretion of tumor necrosis factor-alpha (TNF-α) in Rheumatoid arthritis mice, thereby reducing cartilage and bone destruction.^57^ Matrix proteins like Osteopontin (OPN) and Osteocalcin (OCN) are involved in bone remodeling and mineralization and can be modulated by HDAC inhibitors.^58^ Additionally, lipid signaling molecules are also secreted and can play roles in intercellular signaling, and HDAC inhibition was found to mitigate lipid homeostasis defects by selectively targeting glycerophospholipid metabolism and reducing the accumulation of cholesteryl esters.^59^ Given the broad range of bioactive factors induced by HDAC inhibitors, it is important to explore the mechanisms through which these factors are delivered to target cells. One such mechanism is through EVs. These EVs have therapeutic effects in several diseases, including liver/renal failure, myocardial infraction, ischemic diseases, osteonecrosis, traumatic brain/spinal cord injury, and chronic cutaneous wounds. For instance, Mytidou et al. reported that skeletal muscle-derived EVs can either facilitate local communication between muscle tissues or enter the bloodstream for distant delivery of their cargo.^60^ As known, evidence suggests that histone acetylation in skeletal muscle cells enhances their biological functions and amplifies their therapeutic effects. Our study indicates that TSA treatment enriches the RNA content of EVs from myoblasts, leading to increased transcriptional activity. When administered in vivo, these EVs tend to accumulate more in bone tissues and protect against bone loss, indicating a potential for targeted therapeutic delivery. Besides, the increased secretion of EVs under conditions of histone acetylation, coupled with their enhanced uptake by hBMSCs, suggests an augmentation in their biological potency. Furthermore, we found that EVs from TSA-treated myoblasts promote proliferation, osteogenic differentiation, and migration in hBMSCs, underscoring the therapeutic potential of histone-acetylated muscle-derived EVs in alleviating bone loss.

Current evidence underscores the multifaceted role of skeletal muscle as not only the primary locomotor tissue but also as a pivotal endocrine regulator orchestrating metabolic homeostasis in diverse bodily tissues. While several studies have reported the osteogenic and bone-forming properties of EVs derived from young muscles, our investigations did not unveil a discernible protective effect against bone loss. Moreover, we observed an enhancement in the osteogenic potential of muscle-secreted EVs upon HDAC inhibition, suggesting a potential avenue for therapeutic intervention. The variance in our observations and previous findings may be attributed to several factors. Notably, the heterogeneity in EV isolation methodologies and characterization protocols across different investigations introduces variability in the composition and functional attributes of the EV populations under scrutiny, thereby potentially influencing their biological activity and consequential effects on bone mass and osteogenesis.^61^ Besides, disparities in experimental parameters such as cell lineage, cellular age, health status, administration modalities, and animal models^61,62^ can also contribute to the incongruity in outcomes. For instance, our prior investigation elucidated the secretion of exosomes by aged and dysfunctional muscles, which subsequently modulated Th17 cell behavior, resulting in osteoclastogenesis and bone loss through upregulated SPP1 expression in macrophages.^63^ Additionally, the intricate cargo composition of EVs, including but not limited to microRNAs (e.g., miR-873-3p as evidenced in our study), proteins, and other bioactive molecules, exhibits substantial variability contingent upon donor cell type, physiological state, and therapeutic regimen.^27,64^ These constituents are pivotal determinants of the functional repertoire of EVs, thereby exerting diverse influences on bone homeostasis and osteogenesis. In summary, our in-depth analysis of the literature regarding muscle-derived EVs and their effects on bone has enlightened our understanding of the complexity and nuanced nature of EV-mediated communication and its therapeutic potential. In our study, considering the potential influencing factors such as cell species, mice models, intervention conditions, although we didn’t find that EVs from normal HSMM cultures have protective effects on bone loss, we confirmed our findings that targeting HDACs on skeletal muscle could mimic the benefits of exercise and improve the therapeutic potential of muscle-secreted EVs on bone loss.

The exchange of cellular materials through EVs, including miRNAs, plays a crucial role in intercellular communication.^30,65^ Nie et al. discovered that skeletal muscle-derived EVs promote angiogenesis by regulating endothelial cell functions. They identified several angiogenic miRNAs in skeletal muscle-derived EVs and discovered that miRNA-130a and reactive oxygen species-activated nuclear factor-κB signaling were potentially responsible for mediating the crosstalk between skeletal muscle and endothelial cells.^66^ While the role of skeletal muscle-derived EVs in transferring specific miRNAs is established, a comprehensive analysis of miRNA profiles in EVs from TSA-treated and untreated HSMM cultures, and a mechanistic study of miRNA-mediated effects in promoting bone formation has yet to be reported. Our study fills this gap by profiling these EVs, revealing a significant increase in miR-873 in EVs from TSA-treated HSMM cultures, with implications for bone formation. While miR-873 has been found to have either tumor-suppressive^33^ or tumor-promoting effects^34^ in different types of cancer, its expression was upregulated by PTH treatment in rat osteoblasts, leading to a reduction in HDAC4 protein levels and subsequently stimulating MMP-13 expression in these cells.^35^ Our study showed that histone acetylation-mediated HSMMs produce EVs containing excessive miR-873-3p, which can be directly transferred to hBMSCs and are pro-osteogenic, proliferative, and migratory, all of which are essential for bone formation. Moreover, we observed decreased bone mass phenotypes in mice treated with EVs purified from miR-873-3p-knockdown HSMMs, suggesting that knockdown of miR-873 in TSA-EVs negated the beneficial effects of TSA-EVs on bone formation. These findings suggest that targeting miR-873 may be a potential therapeutic approach for the treatment of osteoporosis, and further investigation into the mechanisms and their relevance to osteoporosis progression is warranted. In addition to investigating the role of specific miRNAs like miR-873-3p in the EVs derived from HDAC-inhibited skeletal muscle cells, it is important to consider the comprehensive profile of bioactive molecules these EVs may carry. Although our current study did not specifically measure the levels of cytokines and other bioactive molecules in EVs from TSA-treated and untreated myoblasts, existing literature suggests that EVs are complex structures capable of encapsulating diverse bioactive molecules, including metabolites, lipids, proteins, and various RNA species.^67^ Recent studies have highlighted the enrichment of specific proteins in EVs derived from TSA-treated cells. For instance, TSA-treated osteoblast-derived EVs have been found to be enriched with proteins involved in transcriptional regulation, including Ankyrin repeat domain-containing protein 11 (AnkRD11) and KAT8 regulatory NSL complex subunit 3 (Kansl3), which are crucial for chromatin remodeling and transcriptional activity.^68^ Additionally, proteins involved in RNA processing such as pre-mRNA-splicing factor 38B (Prpf38b) and RNA-binding protein 10 (Rbm10) were also enriched, indicating a comprehensive alteration in the epigenetic landscape of recipient cells. Given these considerations, it is plausible that EVs from TSA-treated myoblasts carry a diverse array of cytokines and growth factors known to play roles in osteogenesis and bone remodeling. Cytokines such as transforming growth factor-beta (TGF-β), bone morphogenetic proteins (BMPs), vascular endothelial growth factor (VEGF) and insulin-like growth factor 1 (IGF-1) are critical regulators of bone formation and may be present in these EVs. The synergistic effects of these bioactive molecules likely enhance the overall effectiveness of EVs in stimulating bone formation. Future studies should focus on a detailed proteomic and metabolomic analysis of EVs from TSA-treated and untreated myoblasts to confirm the presence and relative abundance of these cytokines and other bioactive molecules. Such analyses would offer a more thorough understanding of the mechanisms driving the osteogenic effects of these EVs.

To decipher the underlying mechanism of EV-miR-873’s impact on bone formation, we employed bioinformatic analyses, identifying *CNN2* as a key target gene. CNN2, an actin filament-associated regulatory protein, is one of calponin’s three isoforms and exhibits widespread tissue distribution. Initially identified in smooth muscle as a troponin-like protein implicated in muscle contraction regulation, subsequent research has expanded its presence to various non-muscle cells.^41,69^ Predominantly, CNN2 expression is noted in tissues and cells undergoing high mechanical tension, rapid proliferation, or active migration. This includes smooth muscle in hollow organ walls, epidermal keratinocytes, endothelial cells, fibroblasts, lung alveolar cells, and various hematopoietic cells such as myeloid white blood cells, platelets, B lymphocytes, and myoblasts.^42,70,71^ These findings suggest CNN2’s potential involvement in cytoskeleton function and cell motility regulation. Recent inquiries into CNN2 have linked it to cancer biology, with notable expression in breast and prostate cancers, suggesting a role in tumor growth regulation.^72^ Moreover, CNN2 deletion in macrophages has been associated with attenuated development of inflammatory arthritis^73^ and atherosclerosis^74^ in murine models, indicating its significance in mechanoregulation within macrophage-mediated diseases. Interestingly, research on *CNN1*, another calponin isoform, has shown its expression in osteoblastic cell lines and bone tissues.^44^ *CNN1* overexpression in osteoblasts significantly decreased bone mass in adulthood by inhibiting osteoblast migration, proliferation, and mineralization, while also promoting osteoclastogenesis.^45^ In our study, we observed a decrease in CNN2 expression in hBMSCs treated with miR-873 mimics and an increase following treatment with miR-873 inhibitor-transfected myoblasts. Notably, CNN2 inhibition in hBMSCs counteracted the miR-873-inhibitor’s negative impact on cell proliferation and osteogenesis. These findings indicate that CNN2 is a key mediator of miR-873’s effects on osteogenic processes in hBMSCs.

While TSA has shown promising potential as a therapeutic agent,^17^ its administration is associated with a number of serious side effects, including fatigue, nausea, vomiting, and hematological toxicities, which are generally only tolerated in life-threatening conditions such as cancer.^75^ This raises concerns about the feasibility of using TSA for non-life-threatening conditions like osteoporosis or muscle regeneration in clinical settings. To address these concerns, our study proposes the use of EVs derived from TSA-treated myoblasts as an alternative therapeutic approach. EVs are nano-sized particles that can transfer bioactive molecules between cells, thereby modulating the recipient cells’ function.^76^ In our previous publications, our research group has elucidated similar cell-based therapies utilizing cell derivatives.^77^ In this study, by stimulating myoblasts with TSA to produce EVs, we can harness the therapeutic benefits of TSA-induced epigenetic modifications without direct systemic exposure to TSA itself, thus potentially mitigating the associated side effects. Moreover, EV-based therapies offer greater specificity and reduced immunogenicity, as EVs can be engineered to target specific cell types or tissues. This targeted approach not only enhances the therapeutic efficacy but also minimizes the risk of off-target effects and systemic toxicity. Our findings indicate that EVs derived from TSA-treated myoblasts have enhanced osteogenic properties, suggesting their potential use in treating bone-related diseases and improving bone health. Altogether, while TSA has significant therapeutic potential, its side effects limit its application in non-life-threatening conditions. EV-based therapeutics provide a promising alternative, offering the benefits of TSA-induced epigenetic modifications with greater specificity and reduced toxicity. Further research into the clinical application of EVs could pave the way for innovative treatments for osteoporosis and other musculoskeletal disorders.

In addition to investigating the role of EVs derived from HDAC-inhibited skeletal muscle cells, it is crucial to address the direct effects of HDAC inhibitors on bone formation. HDAC inhibitors, including TSA, have been studied for their potential to influence bone metabolism directly. HDACs regulate the acetylation status of histone and non-histone proteins, thereby controlling gene expression and cellular function. In bone tissue, HDACs play a role in osteoblast and osteoclast activity, affecting bone remodeling and formation. Previous studies have demonstrated that HDAC inhibitors can promote osteoblast differentiation and bone formation by enhancing the acetylation of histone proteins, leading to the activation of osteogenic genes. For instance, HDAC inhibition has been demonstrated to induce osteoblast differentiation by activating the extracellular signal-regulated kinase 1/2 (ERK1/2) MAPK signaling pathway and increasing ALP activity in osteoblasts.^78^ Additionally, HDAC inhibitors can suppress the activity of osteoclasts, the cells responsible for bone resorption, thereby contributing to an overall increase in bone mass and density.^79^ However, the direct application of HDAC inhibitors for bone diseases is limited by their potential systemic side effects, such as nausea and hematological toxicity.^75^ This is where the use of EVs offers a promising alternative. By harnessing the therapeutic potential of EVs derived from HDAC-inhibited myoblasts, we can potentially achieve similar benefits without the associated systemic toxicity. EVs can deliver bioactive molecules, including miRNAs and proteins, to target cells in a more controlled and specific manner, reducing the risk of off-target effects. In our study, we demonstrated that EVs from TSA-treated myoblasts, which contain elevated levels of miR-873-3p, can enhance osteogenesis by targeting H2 calponin (CNN2). This indirect approach leverages the benefits of HDAC inhibition while mitigating its direct side effects, providing a safer and potentially more effective therapeutic strategy for promoting bone formation. In conclusion, while HDAC inhibitors directly enhance bone formation by promoting osteoblast differentiation and inhibiting osteoclast activity, their systemic side effects limit their clinical application. Our study proposes an alternative approach using EVs derived from HDAC-inhibited myoblasts to achieve similar therapeutic outcomes with reduced toxicity, highlighting the translational potential of this novel strategy.

In conclusion, our study primarily focused on the evaluation of therapeutic strategies using TSA-induced EVs from HSMMs to promote bone formation. The key findings demonstrate that these EVs significantly enhance osteogenesis in vivo, providing a potential non-exercise-based therapeutic approach for osteoporosis. While we identified miR-873-3p as a critical factor in mediating these effects, the detailed molecular mechanisms warrant further investigation. Future research will delve into the pathways of histone modification, EV biogenesis, and miRNA packaging, which are essential for understanding the full therapeutic potential of TSA-induced EVs. This study not only provides insights into muscle-derived EVs as therapeutic agents but also suggests a new method to mimic exercise benefits in disease management. The FDA-approved status of various HDAC inhibitors aligns with our discoveries, offering potential for rapid clinical translation in osteoporosis treatment.

## Materials and methods

### Animal care and study approval

C57BL/6 J mice were sourced from SPF (Beijing) Biotechnology Co., Ltd. The animals were kept in a pathogen-free environment at PLA General Hospital, with a 12-hour dark/light cycle and unrestricted access to food and water. All procedures were approved by the PLA General Hospital Ethics Committee (Approval No. 2022-X18-11).

### Cell culture and reagents

Human Skeletal Muscle Myoblasts (HSMMs) were sourced from Lonza (CC-2580) and cultured in DMEM/F12 Glutamax (Gibco) with 10% FBS and 1% penicillin/streptomycin. The medium was changed every 2 days. At 90% confluence, differentiation was induced by switching to 2% horse serum. For EV collection, medium with EV-depleted FBS was used for 2 days before harvesting. Human bone mesenchymal stem cells (hBMSCs) from Oricellbio (Cyagen) were cultured in hBMSC complete medium under 5% CO2 at 37 °C, with medium changes every 2 days. Cells were collected at 90% confluence using 0.25% Trypsin-EDTA (Gibco) for 3–5 min at 37 °C.

### Cell viability and morphology assessment

HSMMs were seeded in a 96-well plate with basal medium and incubated for 24 h. The medium was then refreshed, with or without TSA (25, 50, 100 nM; Sigma-Aldrich, UK). After 4 h at 37°C, AlamarBlue reagent (Thermo Scientific, UK) was added, and fluorescence was measured using a SPARK spectrophotometer (TECAN, CH) at 540/590 nm. HSMM morphology was also examined using calcein-AM staining and visualized with an EVOS fluorescent inverted microscope (Thermo Scientific, UK) under the same conditions.

### HDAC activity and H3K9 histone acetylation

Cells were cultured in 96-well plates for 24 h, then treated with or without TSA (25, 50, 100 nM) in fresh medium. HDAC activity was measured at 24 and 72 h using a fluorometric assay kit (BioVision, UK), following the manufacturer’s protocol. After incubation with reaction mix and lysine developer, fluorescence was recorded at 368/442 nm and normalized to DNA content. H3K9 acetylation was assessed with the EpiQuik™ In Situ Histone H3-K9 Acetylation Assay Kit (Epigentek, USA), and absorbance was read at 450 nm, also normalized to DNA. DNA content was quantified using the Quant-iT PicoGreen DNA assay (Invitrogen, UK) after cell lysis. Fluorescence was measured at 480/520 nm.

### EdU staining

To assess cell proliferation, we utilized an EdU staining procedure following the instructions provided by the EdU kit (Beyotime, C0078). Cells were exposed to the EdU working solution for 2 h and then fixed with 4% paraformaldehyde for 15 min. After fixation, cells were washed three times with wash buffer and subsequently permeabilized at room temperature for 10 min. The click additive solution was applied to the cells for 30 min in the dark. Lastly, DAPI staining was performed, and the samples were observed using a confocal laser scanning microscope (Nikon A1, version 5.20.00).

### Myogenic differentiation and staining

Human skeletal muscle myoblasts were cultured on cover slips in 6-well plates and then fixed with Fixx solution for 15 min. After permeabilization in 0.1% Triton X-100, cells were blocked with 3% BSA to prevent non-specific binding. They were incubated overnight with a primary antibody (MYL2, 1:140) at 4°C, followed by a secondary antibody (Thermo Fisher Scientific, #A-21206) for 1 h. Nuclei were stained with DAPI. Immunofluorescence was captured using bright field and confocal microscopy, with images processed using Fiji software. The myofusion index was calculated by the ratio of nuclei within myotubes to total nuclei, and myotube diameter and area were measured from random myotubes per image.

### Osteogenic differentiation assay

To induce osteogenic differentiation, hBMSCs were plated in 12-well or 24-well plates at 1 × 10^4^/cm^2^. After 24 h, the growth medium was replaced with osteogenic medium containing MEM, 10% FBS, 100 IU/ml penicillin/streptomycin, 100 nM dexamethasone, 0.2 mM ascorbic acid, and 10 mM β-glycerophosphate. The cells were cultured for 21 days, with medium changes every 3 days.

### Alizarin red staining and quantification

To evaluate cell mineralization, Alizarin red staining was performed. After washing hBMSCs with PBS, they were fixed with 4% formaldehyde for 20 min. Following another PBS wash, cells were stained with 1% Alizarin red for 30 min and then washed with PBS. Calcium nodules were visualized with an inverted microscope. For quantification, the stained samples were treated with 10% hexadecylpyridinium chloride, and the optical density was measured at 562 nm using a microplate reader.

### Alkaline phosphatase staining and activity measurement

To evaluate osteogenesis, cells were first fixed with 4% formaldehyde for 20 min and subsequently stained using the BCIP/NBT Kit (Beyotime, China). Staining was visualized with an inverted microscope.

### Isolation and purification of EVs

EVs were isolated from the conditioned culture medium using a standardized protocol designed to ensure the purity of the EV preparations. To minimize the potential presence of TSA or other small molecules in the isolated EVs, we followed a series of sequential centrifugation and ultracentrifugation steps. Initially, the conditioned medium was centrifuged at 300×*g* for 10 min to remove cells and cellular debris. The resulting supernatant was then subjected to a series of centrifugation steps at increasing speeds (1200 × g for 20 min and 10,000 × g for 30 min) to further eliminate large cellular fragments and debris. Subsequently, the supernatant was filtered through a 0.22 μm pore-size filter to remove particles larger than EVs. To ensure thorough washing and pelleting of EVs and minimize the potential presence of TSA, the collected EV-enriched supernatant was subjected to ultracentrifugation at 100,000 × g for 70 min. The pelleted EVs were resuspended in PBS (Gibco, 11965092), followed by a final ultracentrifugation step to pellet the EVs once more. The resulting purified EV pellet was resuspended in PBS for downstream analysis, including characterization and functional studies.

### Characterization of EVs

EVs were analyzed by transmission electron microscopy (TEM) using a CRYO ARM™ 200 (JEOL Ltd.). Briefly, EV samples were applied to a copper grid, stained with 2% uranyl acetate (HEAD Biotechnology Co., Ltd.) for 2 min, and then dried. For size and concentration analysis, EVs were diluted and examined using the NanoSight LM10-HS system (Malvern), with data analyzed through NTA3.4 software.^80^

### Western blot analysis

EVs were lysed with RIPA buffer, and the protein concentration was assessed using a BCA assay. Proteins were then prepared by heating with SDS-PAGE loading buffer, separated on a 4-20% Bis-Tris gel, and transferred to PVDF membranes. Membranes were blocked, incubated with primary antibodies overnight, then washed and probed with secondary antibodies. Detection was achieved using Clarity Western ECL Substrate, and protein bands were visualized with a gel documentation system and analyzed using ImageJ. Antibodies used included Anti-CD9, Anti-CD81, and Anti-ALIX from Abcam, with a Goat Anti-Rabbit IgG H&L (HRP) secondary antibody.

### Biodistribution of EVs

To track EV distribution, 100 μg of DiR-labeled EVs (D12731, Invitrogen) were injected into the tail vein of mice in a 200 μL PBS solution. Fluorescence imaging was performed at various time points: 0 h, 1 h, 6 h, 12 h, 24 h, 48 h, 72 h, 96 h, and 120 h using the IVIS Spectrum Imaging System (PerkinElmer). At 24 h post-injection, tissues were collected for fluorescence analysis. Fluorescence quantification was conducted using IVIS Living Image software.

### EVs uptake by hBMSCs

Fluorescent labeling of UN-EVs and TSA-EVs was carried out following the manufacturer’s instructions. EVs (50 μg/ml) were labeled with PKH26 (Solarbio, D0030) as previously described.^81,82^ After co-culturing with hBMSCs for 24 h, the PKH26-labeled EVs were observed using a confocal laser scanning microscope (CLSM) with the associated software (A1, version 5.20.00, NIKON).

### OVX-induced osteoporotic mouse model

Female C57BL/6 mice were kept in standard housing with a 12-hour light/dark cycle and allowed free access to food and water. At 12 weeks of age, the mice underwent either ovariectomy (OVX) or sham surgery.^83^ At specified time points, OVX mice were euthanized, and bilateral femurs and tibias were collected for subsequent analysis.

### Animal models and treatments

To investigate the therapeutic effects of TSA-EVs on bone loss, twelve-week-old OVX mice were randomly assigned to receive different treatments. EVs from TSA-treated HSMM cultures (TSA-EVs), untreated HSMM cultures (UN-EVs), or PBS were injected intravenously at a dose of 100 μg per mouse every two days for eight weeks. To investigate the specific role of miR-873-3p in bone regeneration, OVX mice were divided into five groups: WT-EV (EVs from untreated HSMMs), KD-EV (EVs from miR-873-3p knockdown HSMMs), OE-EV (EVs from miR-873-3p overexpressing HSMMs), TSA-WT-EV (EVs from TSA-treated WT HSMMs), and TSA-KD-EV (EVs from TSA-treated miR-873-3p knockdown HSMMs). Each group received intravenous injections of 100 μg EVs every two days for eight weeks. To compare the effects of TSA treatment and exercise on EV content and function, OVX mice were divided into three groups: Non-Exercise (control), Exercise, and TSA-HSMM-EV treatment. The Exercise group underwent standardized treadmill running every two days for eight weeks, while the TSA-HSMM-EV group received intravenous injections of 100 μg TSA-HSMM-EVs every two days for eight weeks.

### Microcomputed tomography analysis

Bone mass and microarchitecture were evaluated using microcomputed tomography (micro-CT) with the Inveon MM system (Siemens, Munich, Germany).^84^ Specimens were scanned at an effective pixel size of 8.89 μm, with parameters set to 60 kV voltage, 220 μA current, and 1500 ms exposure time per rotation step. The imaging involved 1536 slices with an 8.89 μm voxel size in all directions. Three-dimensional (3D) images were reconstructed from the two-dimensional slices, and parameters including bone mineral density (BMD), bone volume/total volume (BV/TV), trabecular number (Tb.N), bone surface area/bone volume (BS/BV), trabecular separation (Tb.Sp), and trabecular thickness (Tb.Th) were analyzed using Inveon Research Workplace (Siemens).^85^

### Bone immunofluorescence staining

For femur immunofluorescence staining, samples were fixed in 4% paraformaldehyde at 4 °C for 12 h, then decalcified in 15% EDTA. After washing, they were immersed in 30% sucrose, embedded in OCT, and cut into 10 μm sections. Sections were permeabilized with 0.5% Triton X-100 and blocked with goat serum (1:10) for 1 h. They were then incubated overnight at 4°C with primary antibodies against OSX (1:100, sc-393325, Santa Cruz Biotechnology) and OPN (1:100, AF7665, Beyotime). Following washing, sections were treated with goat anti-mouse IgG H&L Alexa 488 (1:100, ab150113, Abcam) as the secondary antibody. Finally, nuclei were stained using antifade mounting medium with DAPI.

### Bone histomorphometry analysis

The mice were intraperitoneally injected with calcein (20 mg/kg, i.p.) (Sigma, C0875) 10 d before euthanasia, and injected with alizarin-3-methyliminodiacetic acid (30 mg/kg, i.p.) (Sigma, A3882) 3 d before euthanasia. Femurs were isolated, fixed in 4% paraformaldehyde fix solution, and dehydrated. The samples were then embedded to obtain slices of undecalcified bones. The mineral apposition rate (MAR) and bone formation rate/ bone surface (BFR/BS) were calculated using BioQuant software (OSTEO, version v20.8.60, BioQuant).

### MicroRNA sequencing analysis

MicroRNA sequencing of EVs was carried out by Novogene (Beijing, China). EVs were collected through ultracentrifugation from various samples. For each sample, 3 μg of total RNA was used to prepare small RNA libraries with the NEBNext® Multiplex Small RNA Library Prep Set (NEB, USA). Indexing allowed sample identification. Clustering was done using a cBot Cluster Generation System and TruSeq SR Cluster Kit v3 (Illumina). Libraries were sequenced on an Illumina HiSeq 2500/2000 platform with 50 bp single-end reads. Differentially expressed miRNAs were determined with a fold change cutoff of ≥ 1.5.

### MiR-873 target gene prediction and validation

miR-873 target genes were predicted using TargetScan, miRanda, RNAhybrid, and PITA. To confirm that CNN2 is a direct target of miR-873, a dual luciferase reporter assay was conducted. HEK-293T cells were co-transfected with reporter constructs and either a miR-873 mimic or a negative control. After 24 h, luciferase activity was measured with a dual luciferase reporter system (Promega, E1910) and normalized to Renilla luciferase activity.

### MiR-873 and si*CNN2* transfection

Lentiviral vectors carrying miR-873 mimics, inhibitors, and a negative control (NC) were constructed using the LV2 vector (GenePharma, Shanghai, China). Myoblasts at 40–50% confluence were infected with these vectors at a multiplicity of infection (MOI) of 50. Additionally, small interfering RNA targeting CNN2 (siCNN2) and corresponding control siRNAs (siNC) were obtained from GenePharma.

### Co-culture of HSMM and hBMSC

The well inserts with a 0.4 μm poresized filter (Corning) for six-well plates were used following the manufacturer’s instructions. To examine the effects of HSMMs on hBMSCs, cells were co-cultured in the transwell system. Briefly, HSMMs were cultured in the upper chamber, while hBMSCs were cultured in the lower chamber. hBMSCs were cultured in α-MEM medium for 3d, and then supplied with 5 mM β-glycerol phosphate and 50 μg/mL ascorbic acid to induce osteogenic differentiation. HSMMs were cultured in DMEM medium and transfected with miR-873 mimics, miR-873 inhibitor, or their negative control group. The gene expression of miR-873 and *CNN2* were evaluated by qPCR.

### qPCR

Total RNA was isolated using the FastPure Cell/Tissue Total RNA Isolation Kit V2 (Vazyme) according to the manufacturer’s protocol. Complementary DNA was synthesized from 1 μg of mRNA using HiScript III RT SuperMix (Vazyme). RT-qPCR was conducted with ChamQ Universal SYBR qPCR Master Mix (Vazyme) on a Bio-Rad CFX96 system. Data were processed with Microsoft Excel and GraphPad Prism, and gene expression fold changes were calculated using the 2^-ΔΔCq^ method. All experiments were performed in triplicate. Primer sequences are available in Supplementary Table 2.

### MiR-873 target gene prediction and validation

To verify CNN2 as a target of miR-873, a dual luciferase reporter assay was performed. HEK 293T cells were co-transfected with WT- or Mut-CNN2 3′-UTR Luc reporter plasmids and miR-873 mimic or negative control using Lipofectamine PLUSTM (Invitrogen, China). After 48 h, luciferase activity was assessed with a dual luciferase reporter system (Promega) and normalized to Renilla luciferase activity.

### Statistical analysis

Data were analyzed and plotted using GraphPad Prism 8.0.2. Results are expressed as mean ± standard deviation (SD). A two-tailed Student’s t-test was used for comparisons between two groups, while one-way ANOVA was applied for comparisons among multiple groups unless otherwise specified. All experiments were performed on independent biological replicates unless noted. *P* values ≤0.05 was considered as significant. *P ≤ 0.05, **P ≤ 0.01, ***P ≤ 0.001, ****P ≤ 0.0001.

## Supplementary information

Supplementary Materials

Unprocessed Western Blots

Supplementary Figure 4-Original Figure

## Data Availability

The authors will provide the study’s data upon reasonable request. The microRNA sequencing data have been deposited in the NCBI Sequence Read Archive (SRA) under the project number PRJNA1153983, in accordance with the journal’s publication policy.
