# Supplementary material for: Histone deacetylase inhibition enhances extracellular vesicles from muscle to promote osteogenesis via miR-873-3p
Source: Signal Transduct Target Ther. 2024 Sep 30;9:256. doi: 10.1038/s41392-024-01976-0 (PMC11439940; doi:10.1038/s41392-024-01976-0)
Supplement: Supplementary file 1 — Supplementary Materials [file 41392_2024_1976_MOESM1_ESM.docx]

Supplementary Materials for

**Histone deacetylase inhibition enhances extracellular vesicles from muscle to promote osteogenesis via miR-873-3p**

Ming Chen^1,2,#^, Yi Li^1,2,#^, Mingming Zhang^1,2,#^, Siliang Ge^1,2^, Taojin Feng^1,2^, Ruijing Chen^1,2^, Junmin Shen^1,2^, Ran Li^1,2^, Zhongqi Wang^1,2^, Yong Xie^1,2^, Duanyang Wang^3^, Jiang Liu^3^, Yuan Lin^3^, Feifan Chang^1,2^, Junyu Chen^1,2^, Xinyu Sun^1,2^, Dongliang Cheng^1,2^, Xiang Huang ^1,2^, Fanfeng Wu^1,2^, Qinxiang Zhang^1,2^, Pingqiang Cai^4,5^, Pengbin Yin^1,2,*^ , Licheng Zhang^1,2,*^, Peifu Tang^1,2^

Correspondence to: [zhanglcheng218@126.com](mailto:zhanglcheng218@126.com); [yinpengbin@gmail.com](mailto:yinpengbin@gmail.com)

**This PDF file includes:**

Supplementary Materials and Methods

Supplementary Fig. 1-8

Supplementary Tables 1-2

**Other Supplementary Materials for this manuscript include the following:**

Unprocessed western blots

**Supplementary Materials and Methods**

**Myogenic differentiation and staining.** Human skeletal muscle myoblasts were cultured on cover slips in 6-well plates and then fixed with Fixx solution for 15 minutes. After permeabilization in 0.1% Triton X-100, cells were blocked with 3% BSA to prevent non-specific binding. They were incubated overnight with a primary antibody (MYL2, 1:140) at 4°C, followed by a secondary antibody (Thermo Fisher Scientific, #A-21206) for 1 hour. Nuclei were stained with DAPI. Immunofluorescence was captured using bright field and confocal microscopy, with images processed using Fiji software. The myofusion index was calculated by the ratio of nuclei within myotubes to total nuclei, and myotube diameter and area were measured from random myotubes per image.

**Live cell imaging.** Cell motility under various chemical conditions was assessed using a zenCell Owl incubator microscope (innoME GmbH). Recordings were taken every 30 minutes to measure cell coverage, and the counts of adherent and non-adherent cells, as well as the total number of cells per well. Data were analyzed and visualized using zenCELL Owl software version 3.3.

**Biomechanical assays.** To examine femoral biomechanics, a 3-point bending test was employed. The femurs were loaded using a plunger moving at 1.0 mm/min on an Instron 4302 servohydraulic testing machine. Load-deformation curves were captured during testing. The assessment focused on the femur’s mid-diaphysis, and the following parameters were calculated: maximum load, energy to ultimate load, stiffness, Young’s modulus, and breaking energy.

**Osteoclast differentiation assay.** To differentiate osteoclasts, bone marrow cells from the femurs and tibiae of euthanized mice were cultured in α-MEM with 10% fetal bovine serum (FBS) and 1% penicillin-streptomycin. After 24 hours, the medium was replaced with α-MEM containing 30 ng/mL macrophage colony-stimulating factor (M-CSF) to support precursor proliferation. After 3 days, the cells were transferred to a 24-well plate and treated with 30 ng/mL M-CSF and 50 ng/mL receptor activator of nuclear factor kappa-B ligand (RANKL) to induce osteoclast differentiation. The medium was changed every 2 days. After 5-7 days, cells were fixed with 4% paraformaldehyde, stained with tartrate-resistant acid phosphatase (TRAP), and the number of TRAP-positive multinucleated cells was quantified under a light microscope.

**qPCR.** Total RNA was isolated using the FastPure Cell/Tissue Total RNA Isolation Kit V2 (Vazyme) according to the manufacturer's protocol. Complementary DNA was synthesized from 1 μg of mRNA using HiScript III RT SuperMix (Vazyme). RT-qPCR was conducted with ChamQ Universal SYBR qPCR Master Mix (Vazyme) on a Bio-Rad CFX96 system. Data were processed with Microsoft Excel and GraphPad Prism, and gene expression fold changes were calculated using the 2^-ΔΔCq^ method. All experiments were performed in triplicate. Primer sequences are available in Supplementary Table 2.

**
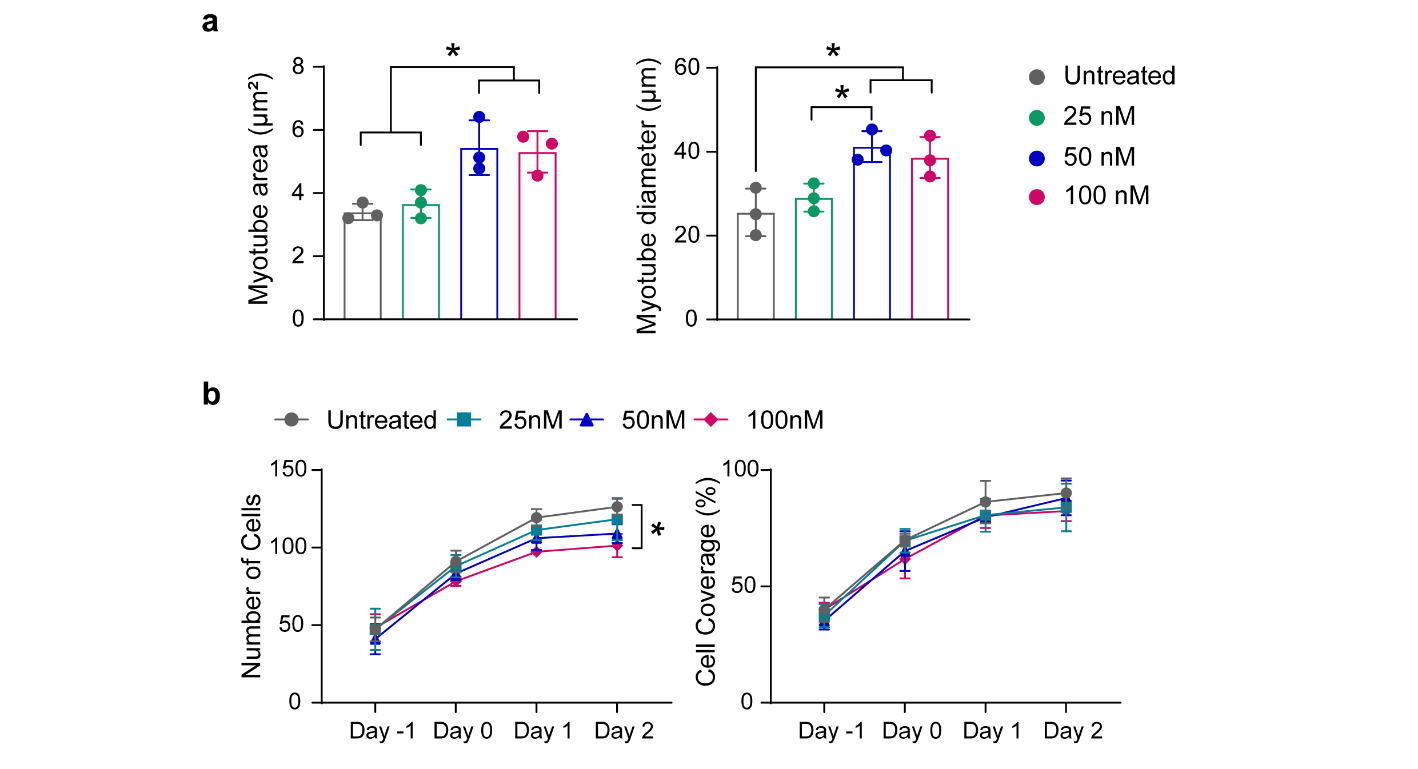
**

**Supplementary Figure 1. The effects of Trichostatin A (TSA) on human skeletal muscle myoblast (HSMM) proliferation and differentiation.** (a) Quantitative analysis of myotube area and diameter during myogenic differentiation (n=3 per group). (b) Cell proliferation of HSMMs assessed by zenCell (n=3 per group). All data are presented as the mean ± SD. *p < 0.05. Statistical significance was determined by one-way ANOVA test (a) and two-way ANOVA test (b).

**
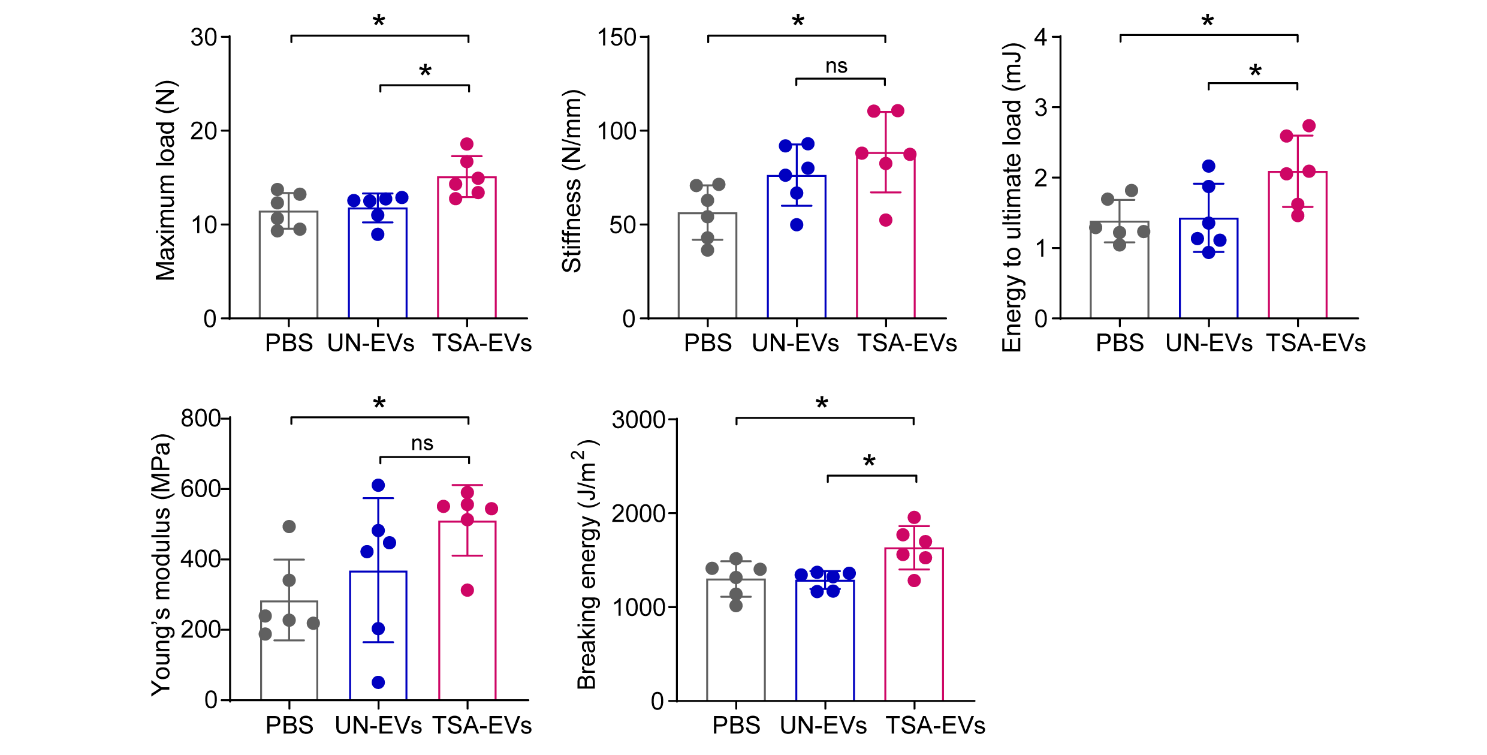
**

**Supplementary Figure 2. TSA-EVs enhance bone mechanical property in ovariectomized (OVX) mice.** Biomechanical analysis of femur, including the maximum load, stiffness, energy to ultimate load, Young’s modulus and breaking energy (n=6 per group). All data are presented as the mean ± SD. *p < 0.05. Statistical significance was determined by one-way ANOVA test.

**
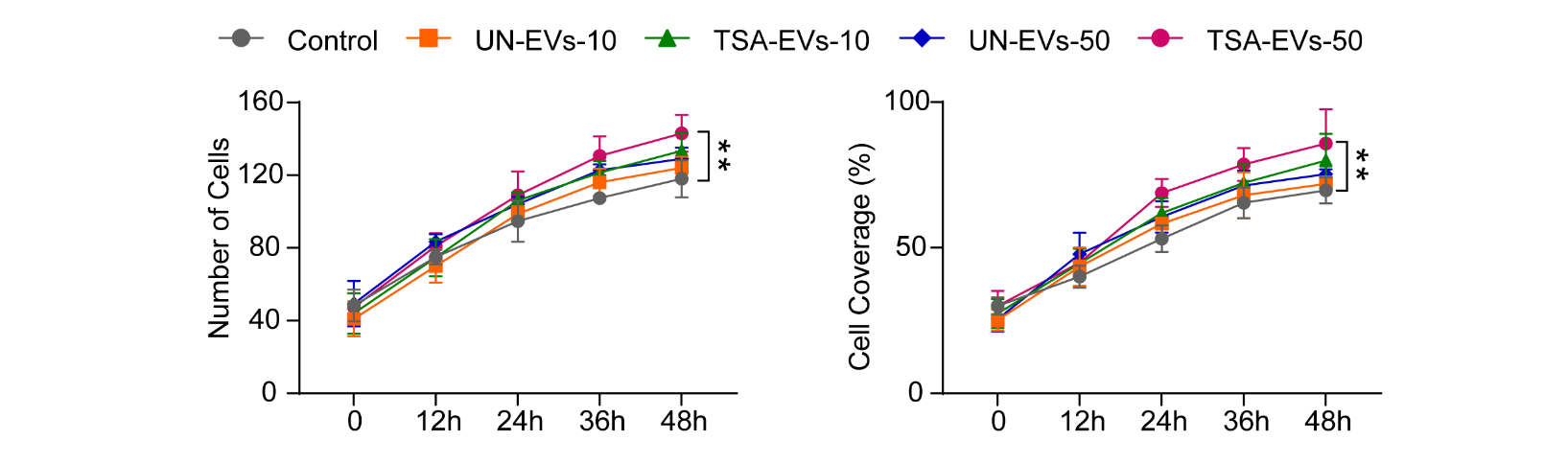
**

**Supplementary Figure 3. The effects of HSMM-EVs on the proliferation of hBMSCs.** Cell proliferation of hBMSCs was assessed by zenCell (n=3 per group). All data are presented as the mean ± SD. **p < 0.01. Statistical significance was determined by two-way ANOVA test.

**
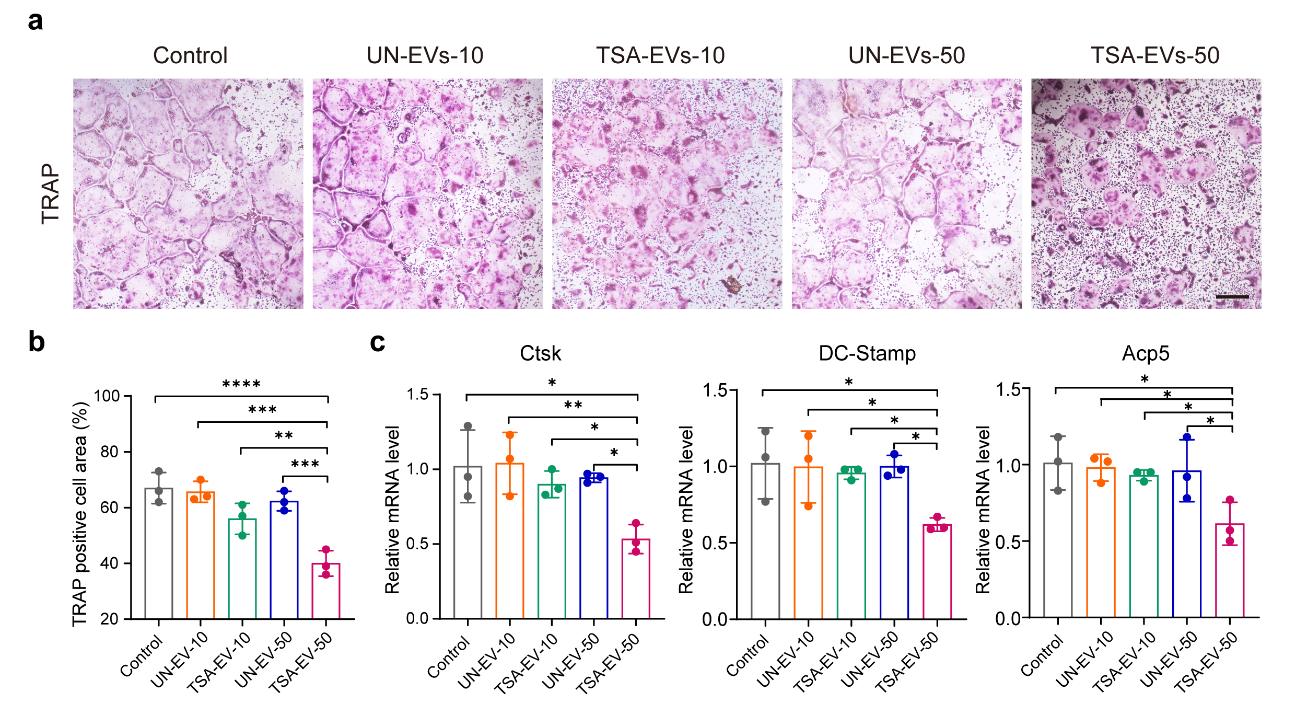
**

**Supplementary Figure 4. The effects of HSMM-EVs on osteoclast formation.**

(a-b) TRAP staining images of osteoclast (a) and analysis of the proportion of osteoclast area (b) after 5 days intervention. Scale bar: 500 μm (n=3 per group). (c) The relative mRNA expression levels of osteoclast-related gene (Ctsk, Dc-stamp and Acp5) (n=3 per group). qPCR results are presented as fold change relative to control. All data are presented as the mean ± SD. *p < 0.05; **p < 0.01; ***p < 0.001; ****p < 0.0001. Statistical significance was determined by one-way ANOVA test.

**
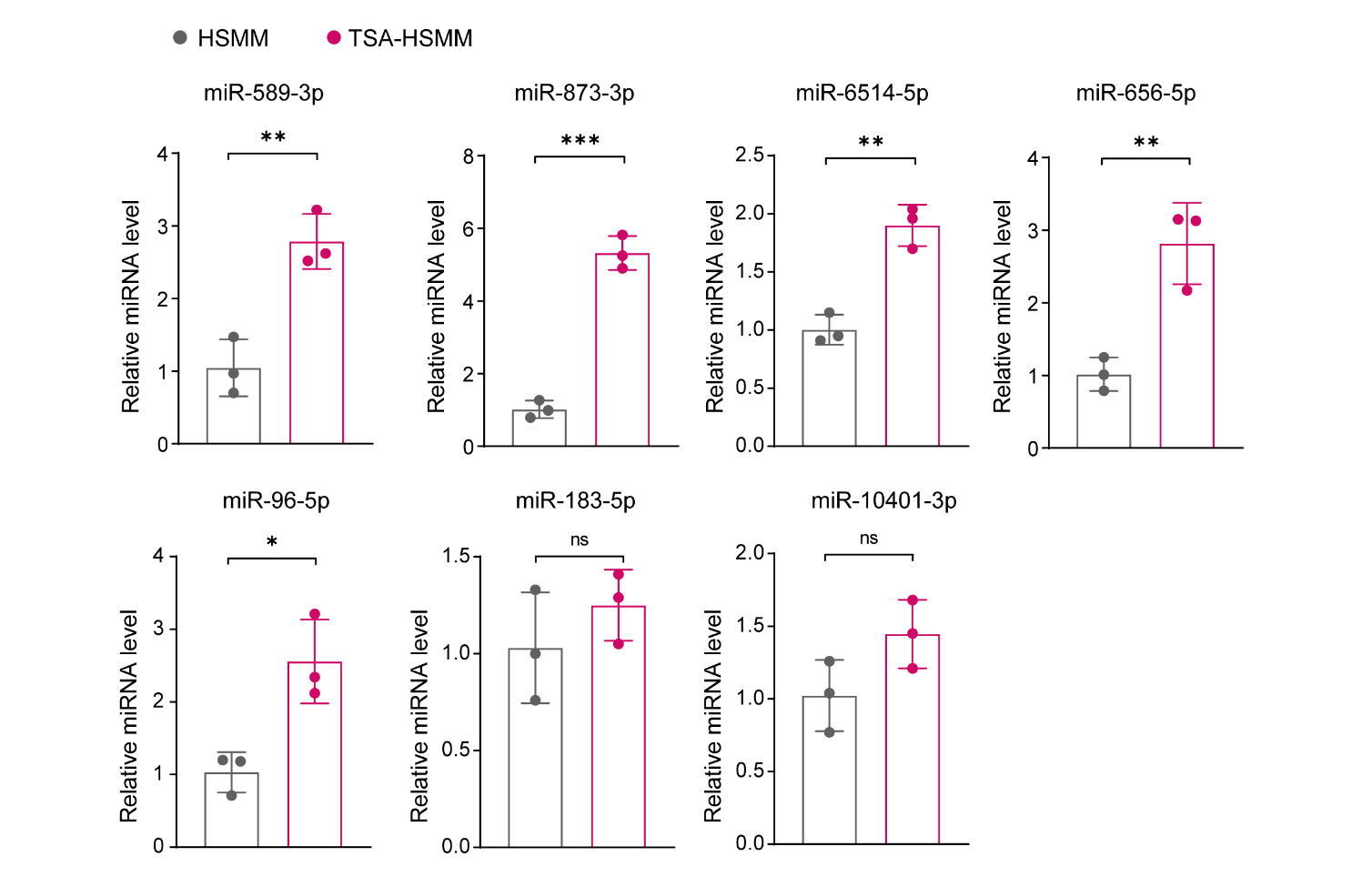
**

**Supplementary Figure 5. Relative expression of miRNAs of interest in vehicle and TSA-treated HSMMs.** Relative expression of miR-589-3p, miR-873-3p, miR-6514-5p, miR-656-5p, miR-96-5p, miR-183-5p and miR-10401-3p in vehicle and TSA-treated HSMMs, as determined by qPCR. qPCR results are presented as fold change relative to control. All data are presented as the mean ± SD. Statistical significance was determined by two-tailed Welch’s t test.

**
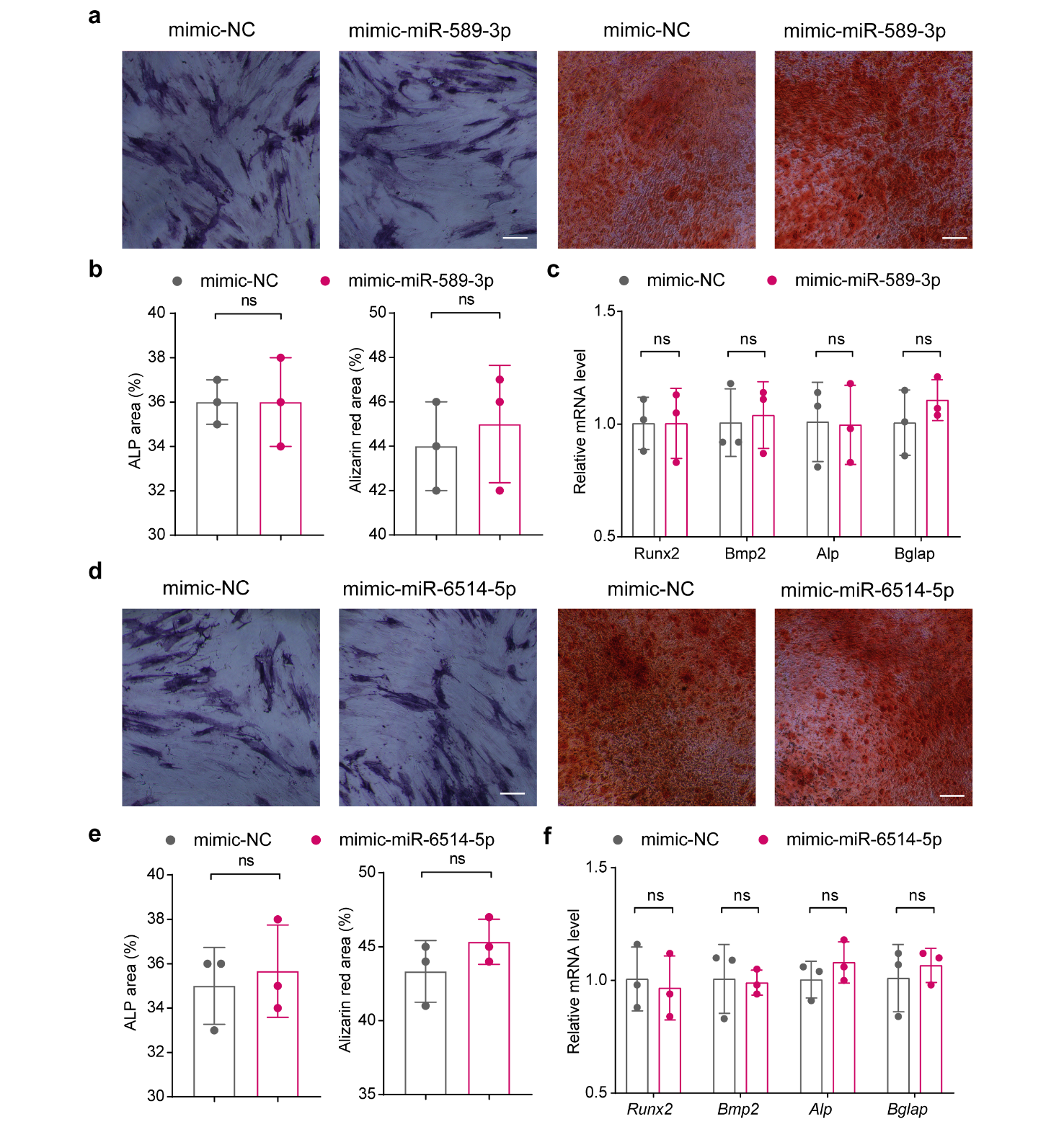
**

**Supplementary Figure 6. Effects of miR-589-3p and miR-6514-5p on osteogenesis.** (a) Representative images of ALP and ARS staining in hBMSCs transfected with miR-589-3p mimics and negative control (mimic-NC). Scale bar = 100 μm. (b) Quantification of ALP positive area (%) and Alizarin red positive area (%) in hBMSCs transfected with miR-589-3p mimics and mimic-NC. n=3 per group. (c) Relative mRNA expression levels of osteogenic markers *Runx2*, *Bmp2*, *Alp*, and *Bglap* in hBMSCs transfected with miR-589-3p mimics and mimic-NC. n=3 per group. (d) Representative images of ALP and ARS staining in hBMSCs transfected with miR-6514-5p mimics and negative control (mimic-NC). Scale bar = 100 μm. (e) Quantification of ALP positive area (%) and Alizarin red positive area (%) in hBMSCs transfected with miR-6514-5p mimics and mimic-NC. n=3 per group. (f) Relative mRNA expression levels of osteogenic markers *Runx2*, *Bmp2*, *Alp*, and *Bglap* in hBMSCs transfected with miR-6514-5p mimics and mimic-NC. n=3 per group. qPCR results are presented as fold change relative to control. All data are presented as the mean ± SD. Statistical significance was determined by two-tailed Welch’s t test.

**
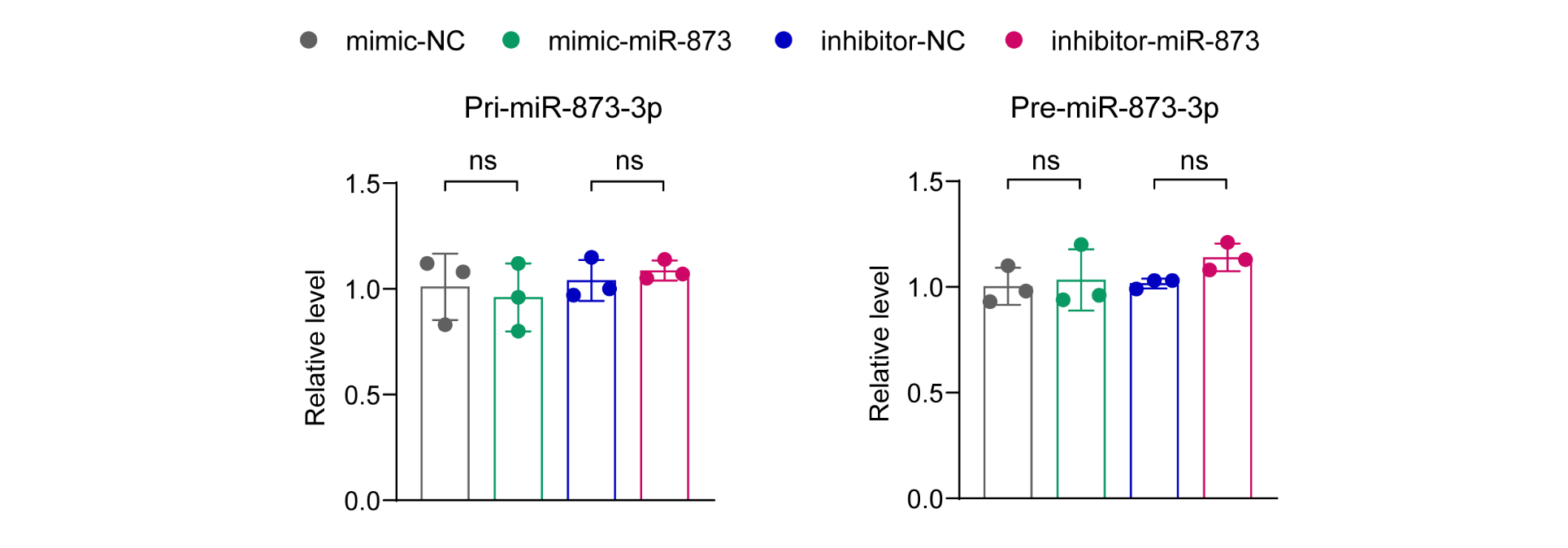
**

**Supplementary Figure 7. Analysis of pri-miR-873 and pre-miR-873 expression in hBMSCs.** The relative expression analysis of pri-miR-873 and pre-miR-873 in hBMSCs after treated by different transfected HSMMs’ EVs (n=3 per group). qPCR results are presented as fold change relative to control. All data are presented as the mean ± SD. ns: no significance. Statistical significance was determined by one-way ANOVA test.

**
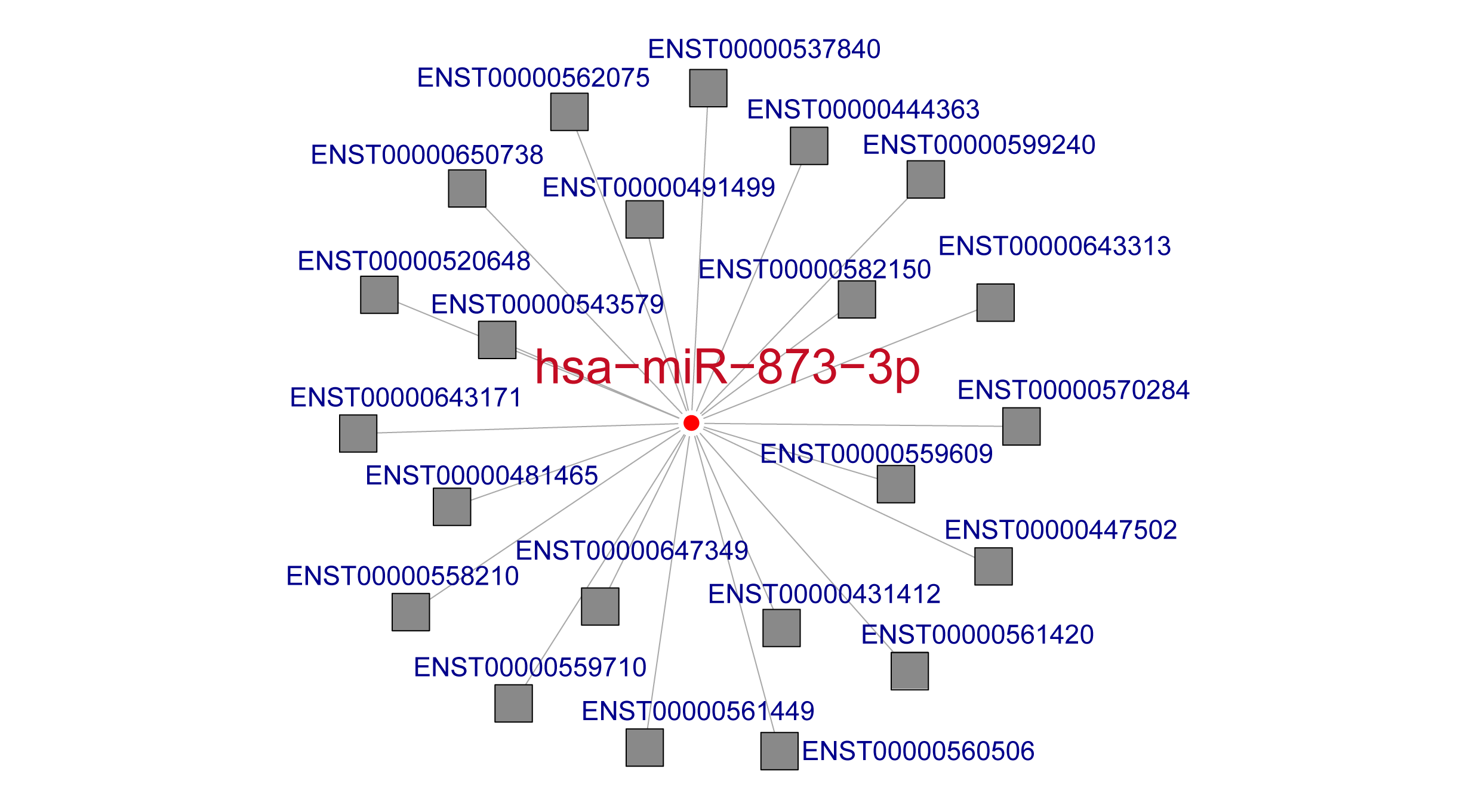
**

**Supplementary Figure 8. Network representation of microRNA hsa-miR-873-3p target interactions.** The central node (red) represents the microRNA hsa-miR-873-3p. Surrounding nodes (blue) with ENST identifiers represent the predicted target genes/transcripts regulated by hsa-miR-873-3p.

**Supplementary Table 1. Differential miRNA expression profile**

| **miRNA** | **TSA-EV**  **readcount** | **UN-EV**  **readcount** | **log2FoldChange** | **pval** |
| --- | --- | --- | --- | --- |
| hsa-let-7f-5p | 21598.28 | 32705.31 | -0.59859 | 0.029515 |
| hsa-miR-1-3p | 1773.551 | 2928.461 | -0.72328 | 0.016693 |
| hsa-miR-100-3p | 24.11564 | 50.26015 | -1.06015 | 0.024393 |
| hsa-miR-10401-3p | 131.9379 | 67.21107 | 0.966831 | 0.019829 |
| hsa-miR-155-5p | 703.1426 | 1068.649 | -0.60327 | 0.048918 |
| hsa-miR-16-2-3p | 65.81051 | 137.2419 | -1.05486 | 0.0232 |
| hsa-miR-183-5p | 48.71663 | 23.84095 | 1.034407 | 0.022222 |
| hsa-miR-20a-5p | 767.4781 | 1515.967 | -0.98138 | 0.016132 |
| hsa-miR-221-5p | 3955.103 | 5967.579 | -0.59334 | 0.035225 |
| hsa-miR-26b-5p | 3384.46 | 5194.3 | -0.61784 | 0.04475 |
| hsa-miR-30e-5p | 4956.4 | 7927.101 | -0.67743 | 0.021714 |
| hsa-miR-32-5p | 149.1612 | 422.2825 | -1.4987 | 0.000512 |
| hsa-miR-369-3p | 615.7725 | 1271.025 | -1.0447 | 0.012349 |
| hsa-miR-374a-3p | 165.4559 | 356.6124 | -1.10534 | 0.009791 |
| hsa-miR-374a-5p | 561.2019 | 1064.487 | -0.92265 | 0.038095 |
| hsa-miR-374b-3p | 12.71087 | 37.85284 | -1.5575 | 0.025439 |
| hsa-miR-374b-5p | 590.7472 | 1094.708 | -0.88904 | 0.044359 |
| hsa-miR-374c-3p | 590.7472 | 1093.978 | -0.88808 | 0.044517 |
| hsa-miR-379-3p | 87.04435 | 140.5524 | -0.68786 | 0.049507 |
| hsa-miR-411-3p | 85.58946 | 164.1101 | -0.93407 | 0.041519 |
| hsa-miR-450b-5p | 269.9924 | 459.0249 | -0.76427 | 0.017874 |
| hsa-miR-4521 | 204.5267 | 524.6631 | -1.35787 | 0.003336 |
| hsa-miR-454-3p | 62.55991 | 125.7482 | -1.00138 | 0.016978 |
| hsa-miR-494-3p | 1874.127 | 3501.016 | -0.90132 | 0.028486 |
| hsa-miR-539-3p | 79.41901 | 193.787 | -1.28155 | 0.009372 |
| hsa-miR-589-3p | 7.27882 | 0 | 5.344617 | 0.002229 |
| hsa-miR-590-3p | 25.32468 | 70.12614 | -1.4577 | 0.015303 |
| hsa-miR-6514-5p | 5.683444 | 0.36496 | 4.031384 | 0.020251 |
| hsa-miR-656-5p | 28.08649 | 10.31246 | 1.446287 | 0.012253 |
| hsa-miR-660-5p | 384.2491 | 675.813 | -0.81326 | 0.027573 |
| hsa-miR-873-3p | 3.990172 | 0 | 4.484168 | 0.02139 |
| hsa-miR-96-5p | 68.90118 | 28.68031 | 1.271202 | 0.006715 |

**Supplementary Table 2. RT-qPCR primer list**

| **Gene** | | **Sequence** |
| --- | --- | --- |
| **qPCR primers** | | |
| *Myod* | F | CTCCAACTGCTCCGACGGCAT |
|  | R | ACAGGCAGTCTAGGCTCGACAC |
| *Myf5* | F | CAGTCCTGTCTGGTCCAGAAAG |
|  | R | GTCCACTATGTTGGATAAGCAATC |
| *Myog* | F | AGTGCCATCCAGTACATCGAGC |
|  | R | AGGCGCTGTGAGAGCTGCATTC |
| *Myh2* | F | GGAGGACAAAGTCAACACCCTG |
|  | R | GCCCTTTCTAGGTCCATGCGAA |
| *Mrf4* | F | CCCTTCAGCTACAGACCCAAAC |
|  | R | TCCTTAGCCGTTATCACGAGCC |
| *Alp* | F | AGCGTGACTTGAAGTGTTGCATG |
|  | R | GAAAGGACCTGGACCACACAGA |
| *Bglap* | F | CGCTACCTGTATCAATGGCTGG |
|  | R | CTCCTGAAAGCCGATGTGGTCA |
| *Col1a1* | F | GATTCCCTGGACCTAAAGGTGC |
|  | R | AGCCTCTCCATCTTTGCCAGCA |
| *Spp1* | F | CGAGGTGATAGTGTGGTTTATGG |
|  | R | GCACCATTCAACTCCTCGCTTTC |
| *Bmp2* | F | TGTATCGCAGGCACTCAGGTCA |
|  | R | CCACTCGTTTCTGGTAGTTCTTC |
| *Runx2* | F | CCCAGTATGAGAGTAGGTGTCC |
|  | R | GGGTAAGACTGGTCATAGGACC |
| Ctsk | F | AGCAGAACGGAGGCATTGACTC |
|  | R | CCCTCTGCATTTAGCTGCCTTTG |
| Dcstamp | F | TTTGCCGCTGTGGACTATCTGC |
|  | R | GCAGAATCATGGACGACTCCTTG |
| Acp5 | F | GCGACCATTGTTAGCCACATACG |
|  | R | CGTTGATGTCGCACAGAGGGAT |
| *Gapdh* | F | GTCTCCTCTGACTTCAACAGCG |
|  | R | ACCACCCTGTTGCTGTAGCCAA |
| miR-873-3p | RT | GTCGTATCCAGTGCAGGGTCCGAGGTATTCGCACTGGATACGACTCCCGG |
|  | F | CGTCAGAACAAATGCCGGTT |
|  | R | AGTGCAGGGTCCGAGGTATT |
| miR-6514-5p | RT | GTCGTATCCAGTGCAGGGTCCGAGGTATTCGCACTGGATACGACGCCAGC |
|  | F | CGCGTATGGAGTGGACTTTCA |
|  | R | AGTGCAGGGTCCGAGGTATT |
| miR-96-5p | RT | GTCGTATCCAGTGCAGGGTCCGAGGTATTCGCACTGGATACGACAGCAAA |
|  | F | GCGTTTGGCACTAGCACATT |
|  | R | AGTGCAGGGTCCGAGGTATT |
| miR-589-3p | RT | GTCGTATCCAGTGCAGGGTCCGAGGTATTCGCACTGGATACGACTCTGGG |
|  | F | CGTCAGAACAAATGCCGGTT |
|  | R | AGTGCAGGGTCCGAGGTATT |
| miR-656-5p | RT | GTCGTATCCAGTGCAGGGTCCGAGGTATTCGCACTGGATACGACTGAACA |
|  | F | GCGAGGTTGCCTGTGAGG |
|  | R | AGTGCAGGGTCCGAGGTATT |
| **miRNA sequence** | | |
| miR-873-3p | F | GGAGACUGAUGAGUUCCCGGGA |
| miR-6514-5p | F | UAUGGAGUGGACUUUCAGCUGGC |
| miR-96-5p | F | UUUGGCACUAGCACAUUUUUGCU |
| miR-589-3p | F | UCAGAACAAAUGCCGGUUCCCAGA |
| miR-656-5p | F | AGGUUGCCUGUGAGGUGUUCA |
