# Supplementary material for: Histone deacetylase inhibition enhances extracellular vesicles from muscle to promote osteogenesis via miR-873-3p
Source: Signal Transduct Target Ther. 2024 Sep 30;9:256. doi: 10.1038/s41392-024-01976-0 (PMC11439940; doi:10.1038/s41392-024-01976-0)
Supplement: Supplementary file 2 — Unprocessed Western Blots [file 41392_2024_1976_MOESM2_ESM.docx]

[**Unprocessed western blots**](https://static-content.springer.com/esm/art%3A10.1038%2Fs41392-024-01930-0/MediaObjects/41392_2024_1930_MOESM1_ESM.pdf)

**
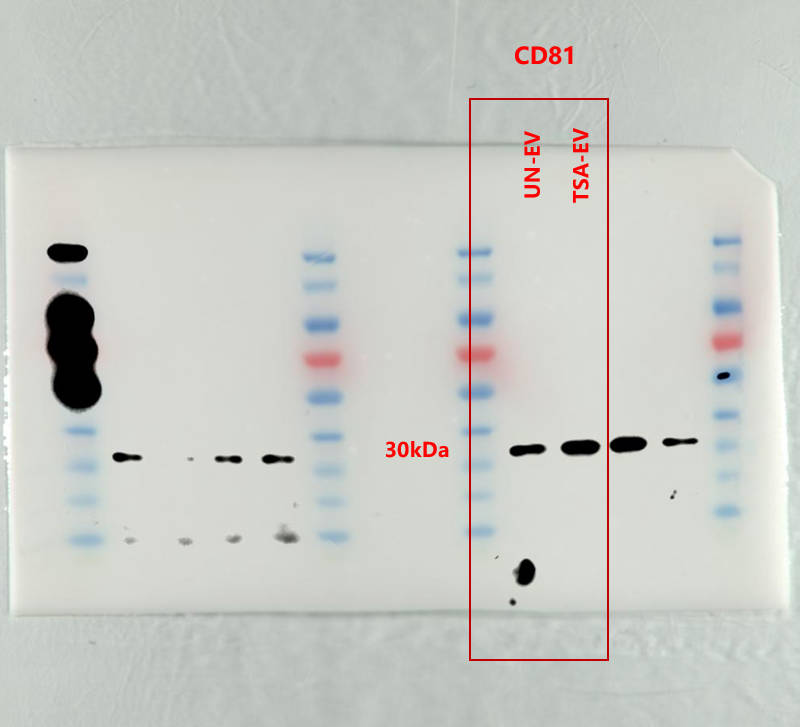
**

**
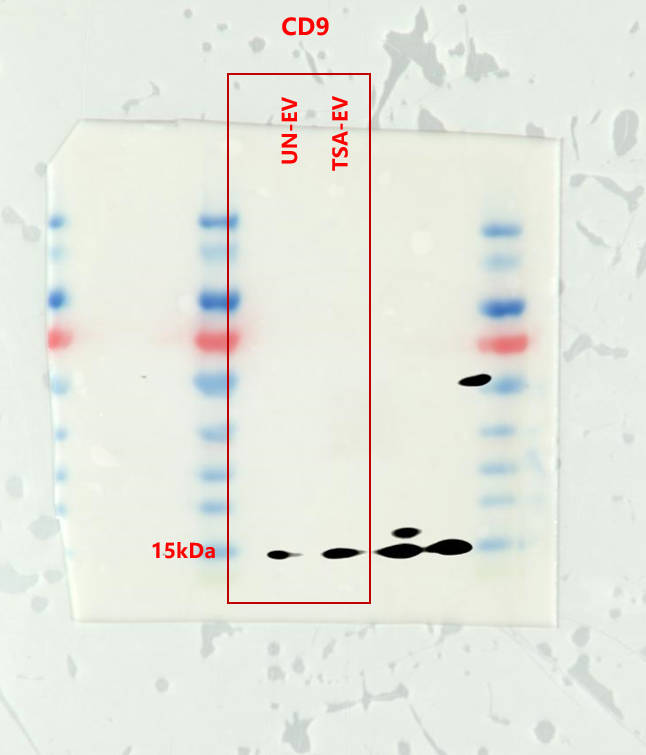
**

**
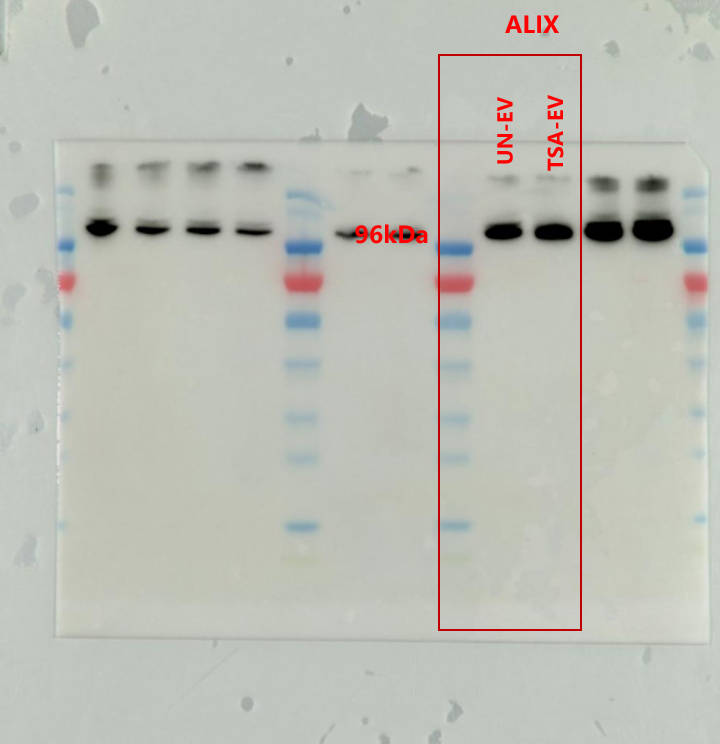
**
