# Supplementary material for: Histone deacetylase inhibition enhances extracellular vesicles from muscle to promote osteogenesis via miR-873-3p
Source: Signal Transduct Target Ther. 2024 Sep 30;9:256. doi: 10.1038/s41392-024-01976-0 (PMC11439940; doi:10.1038/s41392-024-01976-0)

Control-Original Figure

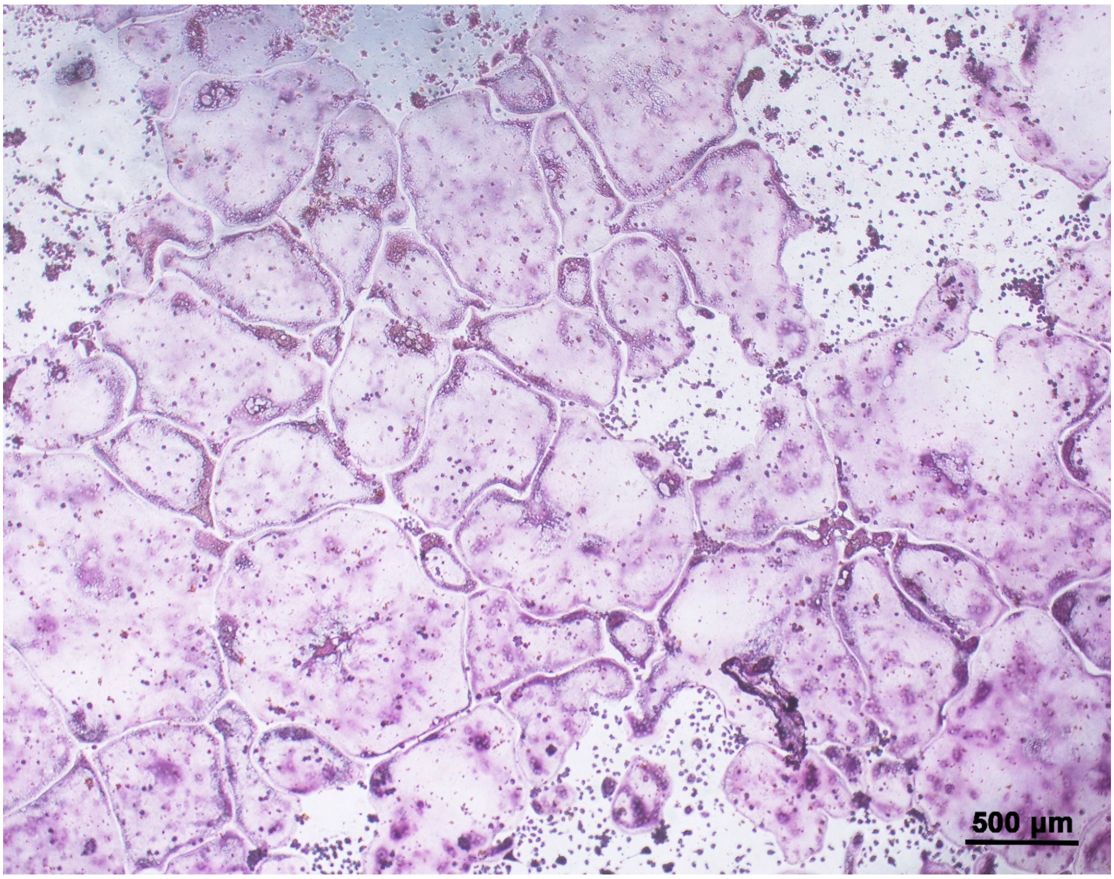

UN-EV-10-Original Figure

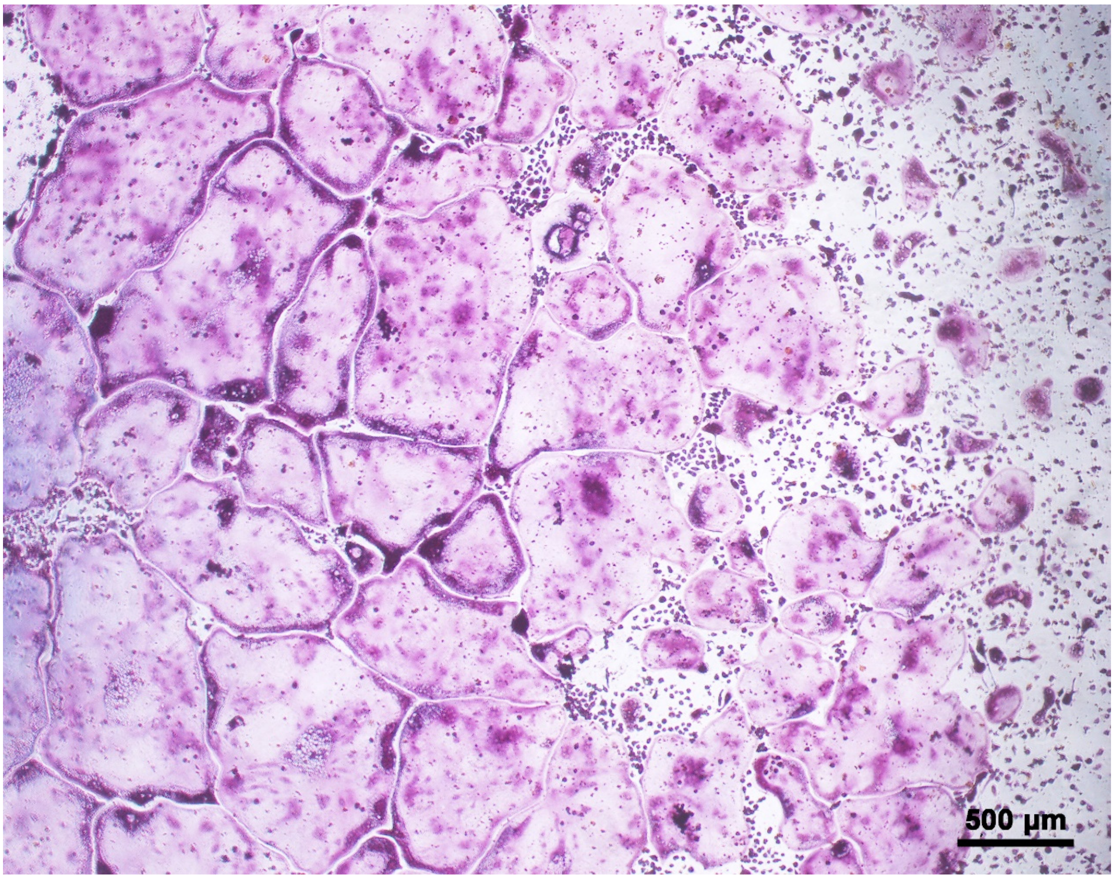

TSA-EV-10-Original Figure

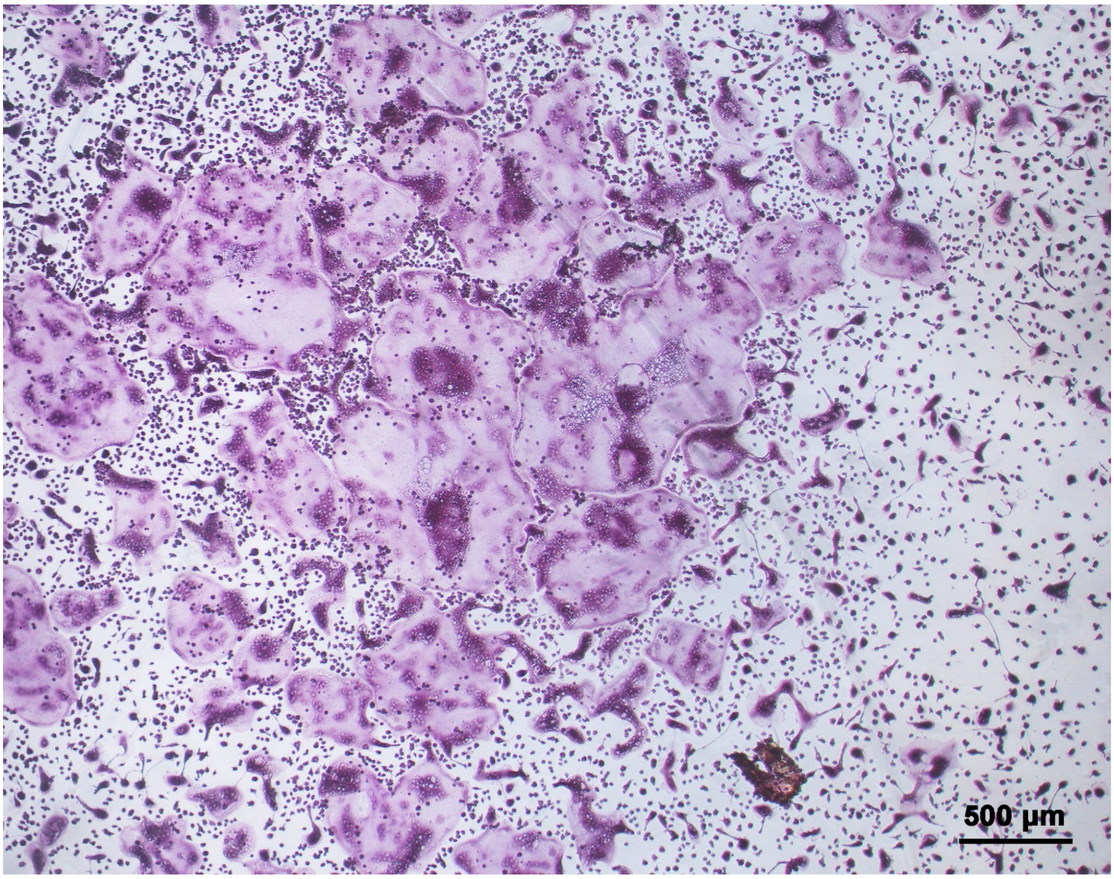

UN-EV-50-Original Figure

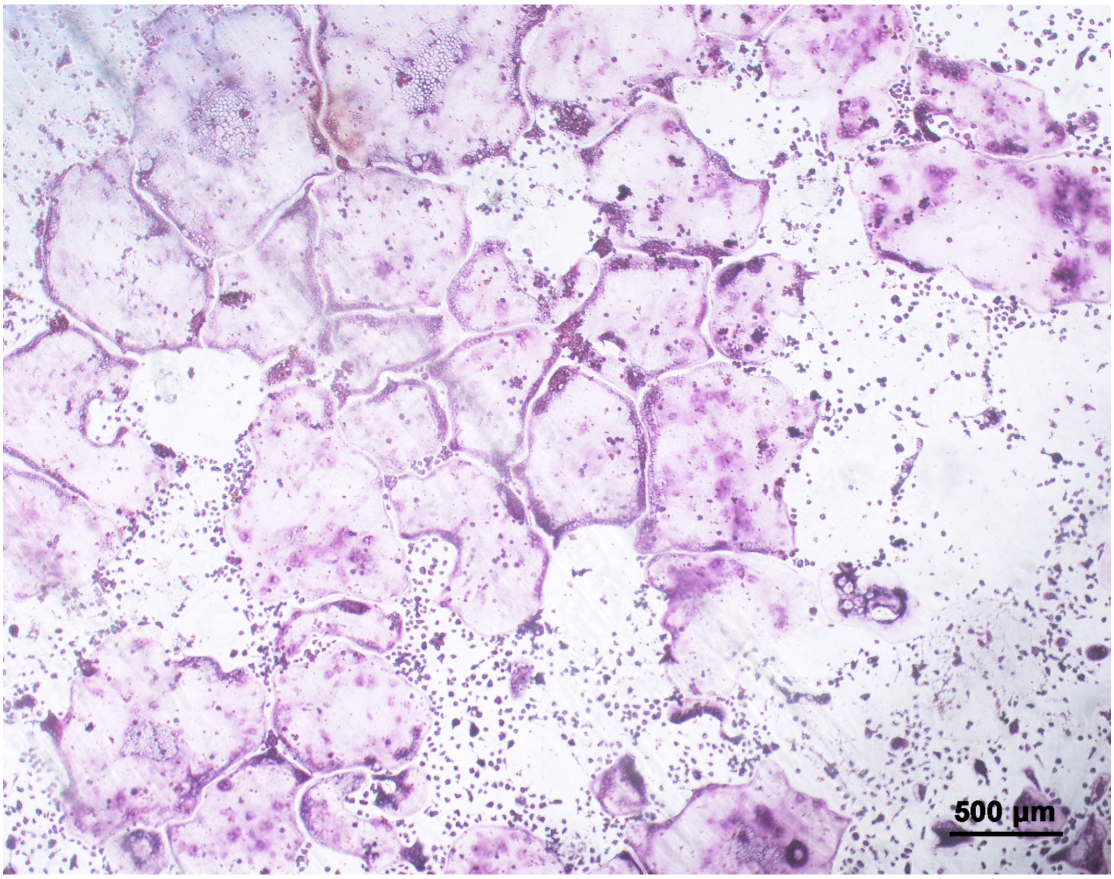

TSA-EV-50-Original Figure

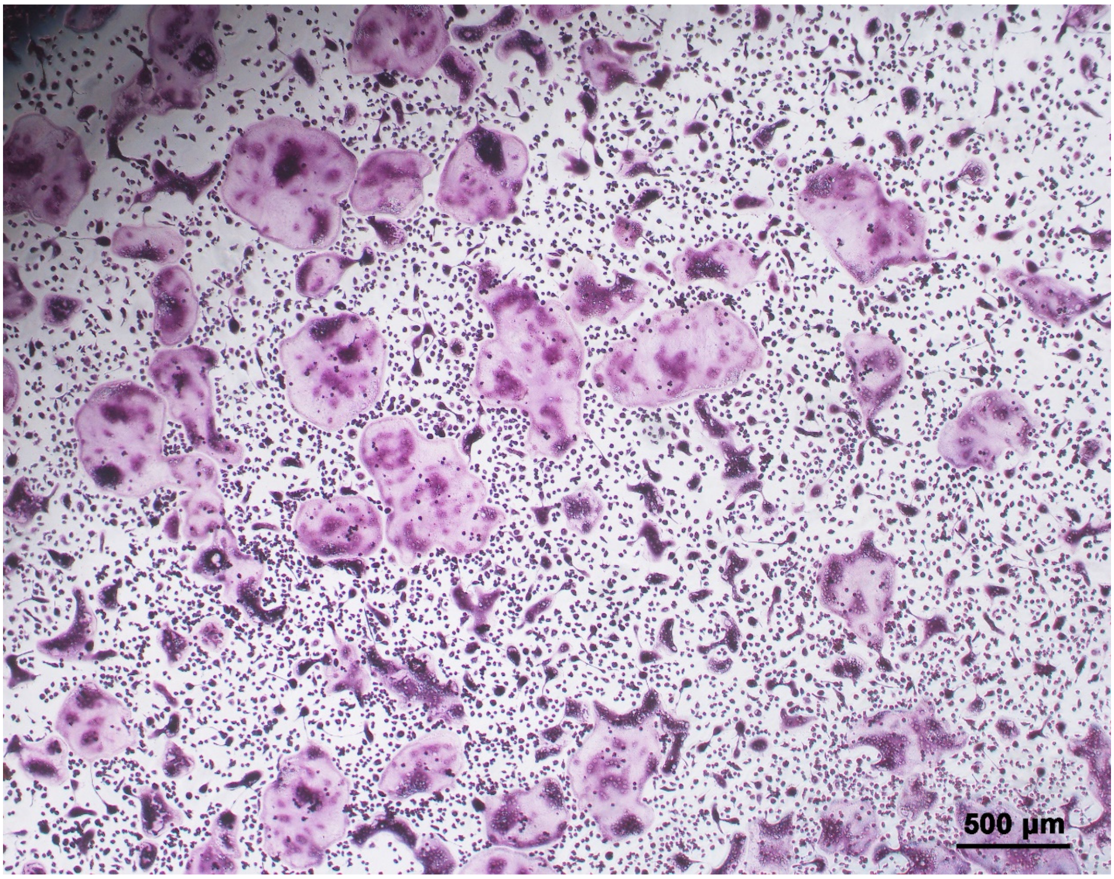

Supplement: Supplementary file 3 — Supplementary Figure 4-Original Figure [file 41392_2024_1976_MOESM3_ESM.pdf]
